# Comprehensive insights into UTIs: from pathophysiology to precision diagnosis and management

**DOI:** 10.3389/fcimb.2024.1402941

**Published:** 2024-09-24

**Authors:** Swathi Sujith, Adline Princy Solomon, John Bosco Balaguru Rayappan

**Affiliations:** ^1^ Quorum Sensing Laboratory, Centre for Research in Infectious Diseases (CRID), School of Chemical and Biotechnology, SASTRA Deemed to be University, Thanjavur, India; ^2^ Nanosensors Laboratory, School of Electrical & Electronics Engineering, Centre for Nanotechnology & Advanced Biomaterials (CeNTAB), SASTRA Deemed to be University, Thanjavur, India

**Keywords:** bacteria, diagnosis, POCT, sensors, UTI

## Abstract

Urinary tract infections (UTIs) are the second most common infectious disease, predominantly impacting women with 150 million individuals affected globally. It increases the socio-economic burden of society and is mainly caused by *Escherichia coli*, *Proteus mirabilis*, *Klebsiella pneumoniae*, *Enterobacter* spp., and *Staphylococcus* spp. The severity of the infection correlates with the host factors varying from acute to chronic infections. Even with a high incidence rate, the diagnosis is mainly based on the symptoms, dipstick analysis, and culture analysis, which are time-consuming, labour-intensive, and lacking sensitivity and specificity. During this period, medical professionals prescribe empirical antibiotics, which may increase the antimicrobial resistance rate. Timely and precise UTI diagnosis is essential for addressing antibiotic resistance and improving overall quality of life. In response to these challenges, new techniques are emerging. The review provides a comprehensive overview of the global burden of UTIs, associated risk factors, implicated organisms, traditional and innovative diagnostic methods, and approaches to UTI treatment and prevention.

## 1 Urinary tract infections: an insightful overview

Infection occurs when the pathogens coercively enter, multiply, and produce toxins in the host for their survival. UTIs, which reduce the quality of life of patients and increase the social burden on society, are the second most common type of infectious disease (after respiratory tract infections) in hospitals and communities (Sahu et al., 2018; Adegun et al., 2019; Danilo de Oliveira et al., 2021; Yang et al., 2022). UTIs are inflammatory reactions of the urinary tract mainly caused by multispecies microorganisms – including bacteria, fungi, and viruses (Amdekar et al., 2011; Seifu and Gebissa, 2018; Wu et al., 2023). Annually, 150 million people worldwide are affected by UTIs in developed and emerging nations (Foxman, 2014; Flores-Mireles et al., 2015).

There is a lack of information on the global incidence and the long-term trends of UTIs, but due to their impact on policy-making and prevention efforts, the Institute of Health Metrics and Evaluation (IHME) conducted a systematic global epidemiological study named Global Burden of Disease (GBD), which used a DisMod-MR Bayesian meta-regression model that quantifies the incidence, mortality, disability, and 87 risk factors for 369 diseases by sex, age, location, and year for 204 countries and territories from 1990 to 2019 (Yang et al., 2022; Zeng et al., 2022). Globally, the total number of incidents, death rates, and DALY (Disability Adjusted Life Year) of UTIs grew significantly from 1990 to 2019, irrespective of gender, geography, and age. Population growth, particularly in the low and middle-income nations, was the driving force behind the sharp increase in the number of infections. The total number of UTI cases increased by 60.40%, from 252.25 million cases in 1990 to 404.61 million cases in 2019. There were 236,790 cases of UTI worldwide in 2019, an increase of 140.18% compared to 98,590 cases in 1990. India had a significant growth in the incident rates, more than doubling between 1990 and 2019, due to the burden on the healthcare system. In 2019, India accounted for 100.8 million incident cases, 55,558 deaths, and 1.59 million DALYs (Yang et al., 2022; Zeng et al., 2022). This review article aims to provide a comprehensive understanding of the various risk factors, and the organisms associated with UTI, conventional and emerging diagnostic methods, along with biomarkers and the management of UTI.

## 2 Understanding UTIs

### 2.1 Anatomy and pathophysiology of UTIs

#### 2.1.1 Structural insights and infection routes

The urinary system is a contiguous hollow organ that includes kidney, ureter, urinary bladder, and urethra. The kidneys are the most important organs, acting as natural filters by clearing the undesired water-soluble debris and reabsorbing components like water, glucose, and amino acids. Through the ureter, urine is directed into the muscular, flexible structure called the urinary bladder until excreted and finally flushed out through the urethra. The urinary tract behaves as a closed system that is impenetrable to pathogens while it is not excreting urine, and the infections occur due to the invasion of the pathogens via the urinary tract, which leads to bladder infection and, subsequently, in some cases to kidney infection with possible kidney damage (Vasudevan, 2014; Hickling et al., 2015). Ascending, hematogenous, and lymphatic are the three ways bacteria use to penetrate and cause UTIs, in which the ascending route is the more prevalent route of infection. Females are more inclined to develop UTIs than males due to shorter urethras, hormonal fluctuations, and proximity to the anus. The progression of infection involves multiple steps. Uropathogens from the gastrointestinal tract or the fecal flora initially attach to the urethra, colonise the bladder, and subsequently spread to the kidneys through the ureters, resulting in cystitis and pyelonephritis, respectively. The enhancement of this route is associated with the use of urinary catheters or spermicidal agents, pregnancy, and ureteral blockage; however, their pathophysiology remains unknown (Foxman, 2002; Davis and Flood, 2011; Mancuso et al., 2023). The descending channel or the hematogenous route (blood-borne route), mainly caused by the *streptococcus* or s*taphylococcu*s spp., comprises less than 5% of UTI cases and typically affects patients with ureteral blockages as well as immunocompromised individuals (Davis and Flood, 2011) (Davis and Flood, 2011). When UTI is severe and complicated, bacteria may enter the bloodstream (bacteremia) and potentially reach other organs or tissues, including the lymph nodes. However, it is not the direct route of UTI progression. Rarely, bacteria from nearby organs may enter the urinary tract through the lymphatics. Retroperitoneal abscesses and severe bowel infections are the conditions connected to the lymphatic pathway (Davis and Flood, 2011).

#### 2.1.2 Prevalent microorganisms triggering UTIs

The healthy urine sample can contain non-culturable bacterial cells and the resident flora is polymicrobial, mainly consisting of *Lactobacillus, Prevotella* and *Gardnerella* in varying proportions. (Siddiqui et al., 2011; Wolfe et al., 2012; Kogan et al., 2015) During an infection, there is a disruption of the flora and severity of the infection correlates with the risk factors in the host (Vasudevan, 2014; Colella et al., 2023; Mancuso et al., 2023). Gram-negative bacteria *E.coli* is the most frequent organism causing UTI. Less common include *Proteus mirabilis, Klebsiella* spp.*, Pseudomonas aeruginosa, Enterobacter* spp., and gram-positive species such as *Streptococcus* spp (Loh and Sivalingam, 2007; Pardeshi, 2020). Uropathogenic *E.coli* (UPEC) accounts for 80–90% and 30–50% of community-acquired and hospital-acquired UTIs respectively, and have been classified into four major UPEC phylogroups (A, B1, B2, and D) based on chromosomal Pathogenicity Islands (PAI) (Terlizzi et al., 2017; Shah et al., 2019; Katongole et al., 2020). The virulence factors determine the pathogenicity of the UPEC and can be broadly classified into two categories, i.e., bacterial cell surface and secreted virulence factors. Fimbriae, flagellum, capsular lipopolysaccharides, and outermost membrane proteins are commonly present in bacterial cell surface virulence factors. The secreted virulence factors are haemolysin and siderophores (Shah et al., 2019). *P. mirabilis*, isolated from 1–10% of all UTIs, is a gram-negative, rod-shaped motile bacteria with peritrichous flagella, characterised by swarming phenomena. Community-acquired urinary tract infections, catheter-associated urinary tract infections (CAUTI), and nosocomial UTIs are also known to be caused by *P.mirabilis*. The virulence factors, which include biofilms, adhesion molecules, urease, proteases, siderophores, and toxins, are correlated to the pathogenesis. These components relate to the interaction of surfaces with bacteria, invasion, injury to host tissues, immune system escape, and iron absorption (Danilo de Oliveira et al., 2021; Hayder et al., 2020; Tabibian et al., 2008; Tabatabaei et al., 2021). *K. pneumoniae*, regarded as the second most prevalent uropathogen, is an encapsulated facultative gram-negative anaerobic bacillus and ferments lactose (Flores-Mireles et al., 2015). The ability of *K. pneumoniae* to attack the immune system and cause a range of diseases depends on virulence factors (Flores-Mireles et al., 2015; Davoudabadi et al., 2023). *P. aeruginosa*, an aerobic, gram-negative, non-fermentative rod-shaped bacterium, is the third most prevalent bacteria associated with hospital-acquired UTIs and accounts for 9% globally (Fu et al., 2013; Heidary et al., 2016). The epidemiology of *P. aeruginosa* infections is influenced by several factors associated with its virulence. The combination of virulence factors influences the severity of an infection. In the general population, *Staphylococcus aureus* accounts for 0.5–6% of urinary tract infections, making it a very infrequent cause of UTIs. Still, they can lead to potentially fatal invasive infections like bacteremia (Xu et al., 2023). Extended hospital stays are a result of MRSA urinary tract infections, which are linked to recent antibiotic usage and urinary catheterization. It is crucial to limit the use of urinary catheterization to necessary situations and remove the device as soon as clinically recommended because there is a strong correlation between catheters and urinary tract infections (Alshomrani et al., 2023).

However, the various bacterial virulent factors act as triggers for initiating infection in the dynamic urinary tract. The lipopolysaccharides on the outer membrane of the gram-negative bacteria are one of the host’s potent inducers of inflammatory responses. Several adhesion proteins found on the cell surfaces of uropathogens are essential for the initial interactions between the host and the pathogen. Biofilms are clusters of microbial cells encased in a polysaccharide-based matrix and are permanently attached to a surface. By positioning themselves to efficiently exploit the available resources and block access to antimicrobials, antibodies, and white blood cells, biofilms provide bacteria with a chance to survive. To remove bacteria from the urinary tract, free urine outflow is required. Urinary stasis provides a more extended period of bacterial adherence and multiplication if bacteria are not mechanically cleared by normal urinary flow due to anatomical or functional conditions (Katongole et al., 2020) (**Table 1**).

**Table 1:** Virulence Genes of Uropathogens.

Virulence Genes Of Uropathogens					
S.No.	Pathogen	Genes	Protein Encoding	Function	Reference
1	UPEC	*fim* operon	Type 1 Fimbriae	Adherence and colonization	(Mancuso et al., 2023; Whelan et al., 2023)
			Type 2 Fimbriae	Adherence and colonization	
		*sfa*	S Frimbriae	Adherence and colonization	
		*dra*	Dr Frimbriae	Adherence and colonization	
		*foc* operon	F1C Fimbriae	Adherence and colonization	
		*pap* operon	Pap Pilli	Adherence and colonization	
		*csg*	Curli	Adhesion, Colonization, Biofilm formation	
		*afa*	Afimbrial Adhesins	Adherence and colonization	(Terlizzi et al., 2017)
		*hlyA*	α-hemolysin	Tissue damage (Pore formation), Dysfunction of the local immune response	
		*cnf 1*	Necrotizing cytotoxic factor 1	Renal invasion	
		*iutA*	Aerobactin	Iron acquisition	
2	*Proteus mirabilis*	*MR/P* operon	Mannose-resistant/Proteus-like Fimbriae	Adherence and colonization	(Oliveira et al., 2021; Hayder et al., 2020; Tabibian et al., 2008; Tabatabaei et al., 2021)
		*uca*	Uroepithelial cell adhesion fimbriae	Adherence and colonization	
		*atf* operon	ATF	Adherence and colonization	(Zunino, 2000)
		*pmfACDEF*	PMF/*Proteus Mirabilis* Fimbriae	Adherence and colonization	(Oliveira et al., 2021; Hayder et al., 2020; Tabibian et al., 2008; Tabatabaei et al., 2021)
		*pmp*	*Proteus mirabilis* P-like pili	Adherence and colonization	(Bijlsma et al., 1995)
		*zapA*	ZapA metalloprotease	Protease activity	(Oliveira et al., 2021; Tabatabaei et al., 2021; Hasan et al., 2021; Tabibian et al., 2008)
		*hpm*	Hemolysin	Toxin generates pores in the target membrane	(Oliveira et al., 2021; Hayder et al., 2020; Tabibian et al., 2008; Tabatabaei et al., 2021)
		*pta*	Proteus toxic agglutinin (pta)	Colonization	
		*ureRDABCEFG*	Urease	Hydrolyze urea to ammonia and Carbon dioxide	
3	*K. pneumoniae*	*fimBEAICDFGH* Operon	Mannose-sensitive type 1 pili	Adherence and colonization	(Wilksch et al., 2011; Sarshar et al., 2020)
		*mrkABCDF* Operon	Type 3 pilli	Adherence and colonization	
			Capsule	Anti- phagocytic Activity	
4	*P. aeruginosa*	*-*	Flagellin	Motility	(Allison et al., 1985)
		*toxA*	Exotoxin A	Tissue necrosis.	(Newman et al., 2017)
		*exoS*	exoenzyme S	Inhibit eukaryotic cell function	
		*lasB*	Elastase	Prteolytic Enzyme act on connective tissue	
		*plcH*	Phospholipase C	Hydrolyzes phospholipids.	
		*-*	Pyoverdine, pyochelin	Siderophores- acquire Iron	
5	*Enterococci* spp.	*ace*	Collagen-binding protein,	Collagen and laminin adhesin	(Haghi et al., 2019)
		*PAI*	Pathogenicity islands,	Virulence factor	
		*asa1*	Aggregation substance	Aggregation, adherence	
		*sprE*	Serine protease	Proteolytic activity	
		*cylA*	Cytolysin	Toxin that aids virulence	
		*esp*	Enterococcal surface protein	Function unidentified	(Toledo-Arana et al., 2001)
		*gelE*	Gelatinase	Peptide hydrolysis	(Haghi et al., 2019)
		*hyl*	Hyaluronidase	Tissue damage	(Golińska, 2013)
6	*Streptococcus* spp.	*uafA*	Uro-adhesion factor A	adherence to the epithelial cells of the human bladder	(Rafiee and Ghaemi, 2023) (de Paiva-Santos et al., 2018)
		*aas*	Hemagglutinin Aas	adherence and autolytic characteristics	
		*ureACD*	urease	development of urinary infectious stones and is necessary for the effective colonization of the kidneys and bladder.	(Xu et al., 2023)
		*dsd*A	D-serine deaminase	survival in the bladder environment	
		*pvl*	Pantone Valentine leucocidin	Toxin	(Baba-Moussa et al., 2008)
		*(fnbPA*	fibronectin binding protein A	adherence	(Baba-Moussa et al., 2008; Goudarzi et al., 2019)
		*clf*	clumping factor	Adhesion to fibrinogen	
		*ebp*	elastin binding protein	Adhesion	
		*lbp*	laminin binding protein	Adhesion	
		*(icaABCD*	polysaccharide intercellular adhesion	adhesion	
		*spa*	staphylococcal protein A	biofilm formation	
		*hla*	hemolysin	osmotic cytolysis, cellular depolarization and the loss of vital molecules	(Aubais aljelehawy et al., 2021)
		*mecA*	PBP2A protein	bacterial cell wall formation) and resistance to some antibiotics	

### 2.2 Categories of UTIs: a differentiation

UTI classification is crucial for medical assessment, research, quality assurance, and education (Smelov et al., 2016). To develop more homogeneous study groups for the evaluation of novel anti-infective medications in clinical trials, the Infectious Diseases Society of America (IDSA) and the European Society of Clinical Microbiology and Infectious Diseases (ESCMID) introduced the concepts of uncomplicated UTI and complicated UTI in 1992 (Wagenlehner et al., 2020). Most individuals with uncomplicated UTIs are healthy, without structural or neurological abnormalities of the urinary system. A complicated UTI is an infection that occurs in conjunction with another condition, such as an abnormality of the genitourinary tract’s structure or function, or the presence of an underlying disease, such as urinary obstruction, a neurological condition that causes urinary retention, renal failure, renal transplantation, pregnancy, or the presence of foreign bodies like calculi, indwelling catheters, or other drainage devices (Flores-Mireles et al., 2015; Pardeshi, 2020). The broad range of symptoms and the heterogeneous nature of the disease have raised concerns that the findings from the clinical studies on patients who were diagnosed with complicated UTI using one set of criteria would not be applicable to patients diagnosed using another set of criteria (Wagenlehner et al., 2020).

The ORENUC classification system developed by the European Association of Urology classifies UTIs based on clinical presentation, risk factors (RFs), and severity. This system classifies adults with uncomplicated UTIs as O (no known/associated RF), R (recurrent UTI RF, but no risk of severe outcome), and occasionally E (extraurogenital RF, with risk of more severe outcome), while complicated UTIs are classified as N (nephropathic disease, with risk of more severe outcome), U (urological RF, with risk of more severe outcome, which can be resolved during therapy), and C (permanent urinary catheter and non-resolvable urological RF, with risk of more severe outcome) (Smelov et al., 2016; Tan and Chlebicki, 2016; Gama et al., 2020; Johansen et al., 2011). Based on the site of infection, UTIs can be classified into urethritis, cystitis, and pyelonephritis (Wagenlehner et al., 2020). Cystitis is an infection in the urinary bladder that can be classified as uncomplicated or complicated (Li and Leslie, 2023).

Pyelonephritis affects the kidneys, can be classified as both uncomplicated and complicated. Uncomplicated cases typically appear with few symptoms and are curable. Pregnant women, people who have had kidney transplants, uncontrolled diabetes, urinary anatomical anomalies, acute or chronic kidney failure, immunocompromised patients, and having acquired bacterial infections from hospitals are all at risk for complicated pyelonephritis (Venkatesh, 2017; Belyayeva and Jeong, 2023).

### 2.3 UTI risk factors: identifying contributors

**Figure 1:**
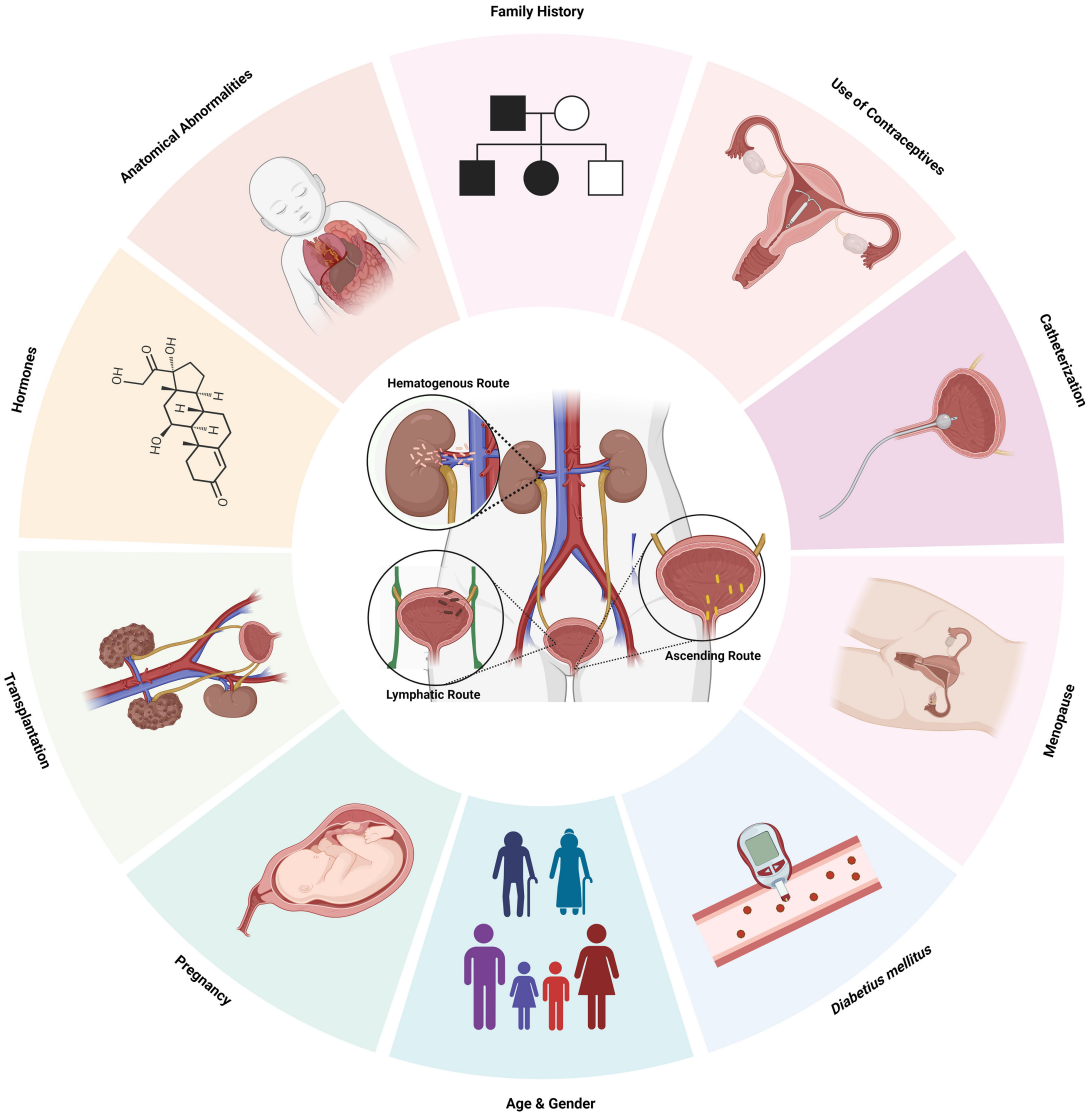
Risk Factors and routes of infection of UTI. *Created Using BioRender.com*.

A clinical examination, record history, and request for more preoperative tests are common procedures to check for any diseases or ongoing treatments that may raise the risk of infection (Marmor and Kerroumi, 2016). UTI classification involves risk variables as a fundamental component. Physicians can effectively modify preventative treatments to lower the likelihood of recurrence by thoroughly understanding the risk factors linked with recurrent UTIs (Storme et al., 2019) (**Figure 1**; **Table 2**).

**Table 2:** Risk Factors of UTI.

	Risk factors														
S. No	Types	Gender	Age	Diabetes	Menopause	Catheterization/Foreign Body	Frequent Sexual Intercourse	Use of Contraception	Family History	Anatomic Abnormalities	Antimicrobials/Antibiotic Use	Hormonal Changes	Transplantation	Pregnancy	Reference
1	Cystitis	+	+	+	+	+	+	+	+	+	+	+	+	+	(Schmiemann et al., 2010; Yoon et al., 2013)
2	Pyelonephritis	+	+	+	+	+	+	+	+	+	+	+	+	+	(Scholes et al., 2005; Bethel, 2012)

#### 2.3.1 Age and gender

Urinary tract infections affect people of all ages, and their frequency rises with advancing age in both genders (Bardsley, 2017; Ünsal et al., 2019). Boys experience the highest prevalence of UTIs in the initial three months after birth, and it is common in girls after 6 months of age, which then holds a steady trend until late childhood and adolescence (Ünsal et al., 2019). UTI occurrence declines in middle age but again rises in elderly persons (Rowe and Juthani-Mehta, 2013; Akhtar et al., 2021). Compared to men, women are more prone to lower UTIs due to the presence of the short urethra as well as the proximity of the urethral opening to the anus and vagina which are considered as the reservoirs of bacteria. Lower UTI usually manifests in men after 60 years due to prostate syndrome.

Age-related immune function changes (progressive T-cell dysregulation and general deterioration of mucosal immunity), urinary incontinence, impaired emptying with residual urine, urethral catheters and instrumentation, obstructive uropathy from prostatic disease in older men, declining estrogen levels, diabetes, kidney stones, and greater exposure to nosocomial and environmental pathogens may contribute to the persistence or recurrent UTI (rUTI)older people (Gavazzi and Krause, 2002; Bardsley, 2017; Hsiao et al., 2020; Martischang et al., 2021; Dutta et al., 2022).

#### 2.3.2 Diabetes mellitus

The risk of UTIs is correlated with more significant duration and severity of diabetes (Chen et al., 2009). Elevated levels of glucose in urine serve as a culture medium to encourage the growth of uropathogens and the decreased immune function, including humoral, cellular, and innate immunity, such as impaired migration, intracellular killing, phagocytosis, or chemotaxis in polymorphonuclear leukocytes (Chen et al., 2009; Wang et al., 2013; Saliba et al., 2015). Patients with type 2 diabetes have a higher incidence of all UTI types. In contrast to people without diabetes, patients with diabetes have lower amounts of IL-6 and IL-8 in their urine (Soo Park et al., 2006; Schneeberger et al., 2014; Saliba et al., 2015).

#### 2.3.3 Menopause

UTI in premenopausal women and postmenopausal women have fundamentally different pathogenesis. *Lactobacillus* spp. colonize the healthy premenopausal vagina and maintain a protective vaginal microbiome, hindering the adherence of the uropathogens by the production of lactic acid from the glycogen produced by the vaginal epithelial cells. The women are vulnerable to developing both primary and rUTIs during menopause due to the drop in estrogen levels that thins the vaginal epithelium and reduces glycogen levels which indirectly increases the colonisation of pathogenic bacteria (Perrotta et al., 2008; John et al., 2016; Caretto et al., 2017; Jung and Brubaker, 2019).

#### 2.3.4 Catheters/foreign body

Catheter-associated urinary tract infection accounts for 70–80% of infections because devices such as indwelling catheters act as the site of infection by introducing opportunistic organisms into the urinary tract at the junction of the catheter collecting tube or at the portal of the drainage bag (Nicolle, 2014). Insertion of the urinary catheter allows the organisms to ascend into the bladder and start to produce symptoms within 25-72 h due to the damage in the uroepithelial mucosa, which exposes new binding sites for bacterial adhesins and also interferes with the host mechanical defences such as incomplete voiding (Garibaldi et al., 1980; Hashmi et al., 2003; Jacobsen et al., 2008). Prolonged catheterization, disconnection of the catheter and drainage tube, and the absence of systemic antibiotic medication are all risk factors for catheter-associated urinary tract infections (John et al., 2016).

#### 2.3.5 Contraceptive

The barrier methods of contraception are more predisposed to causing urinary tract infections than other types. Long-term complications can be avoided with health education on the hygienic and reliable use of family planning techniques (Fihn et al., 1985; Dienye and Gbeneol, 2011). The usage of contraceptives exacerbates the susceptibility of women to urinary tract infections due to the colonisation of the uropathogens in the vagina and periurethral area. Therefore, UTI among those who use barrier contraceptives may result from unhygienic conditions during condom application. The vaginal wall may be damaged by unlubricated condoms, leaving it open to infections. Additionally, it has been proposed that those who utilise barrier methods are susceptible to UTI as they encounter elevated vaginal fluid pH, changes to the normal vaginal flora, and greater rates of introital *E. coli* colonisation (Acton and O’Meara, 1997; Dienye and Gbeneol, 2011). The use of an IUD increases the risk of urinary tract infection, particularly in women who have rUTIs (Fallahian et al., 2005).

#### 2.3.6 Chronicles of UTIs

Intraindividual variations in incidence and severity UTIs with intraindividual variations in incidence and severity are common among females with a genetic history due to the impact of several heritable genes which include *HSPA1B, CXCR1 and 2, TLR1,2,4,5, SIGIRR, TRIF, TRAM, MyD88 TIRAP, VEGF*, and *TGF-1* (Hagberg et al., 1985; Hopkins et al., 1999; Lundstedt et al., 2007; Zaffanello et al., 2010).

#### 2.3.7 Structural or functional anomalies

Vesicoureteral reflux, which mainly affects children, impedes the flow of urine, and reduces the bladder emptying efficiency, which in turn serves as a medium for the uropathogen to adhere and multiply, thus causing an infection (Yuyun et al., 2004; Hickling et al., 2015; Kaufman et al., 2019). The study by Yuyun et al. found that, compared to males without urinary tract infections, men from Cameroon had a much higher prevalence of UTI due to anatomical and functional abnormalities (Yuyun et al., 2004). Ureteral obstruction caused by urinary calculi may serve as a surface for bacteria to attach to and proliferate, thus accounting for rUTIs (Hickling et al., 2015).

#### 2.3.8 Transplantation

More than 75% of kidney transplant recipients acquire urinary tract infections. UTI lowers the quality of life associated with health and may affect transplant function, thereby lowering graft and patient survival. The risk of UTI is increased by recipient-related characteristics such as waiting time before transplantation, female gender, age, or a history of rUTI, diabetes mellitus, and urinary tract abnormalities or by organ factors such as re-transplantation, duplex ureters, and deceased donors and transplantation factors such as Foley catheters, ureteric catheters, transplant malfunction, and rejection. Additionally, the degree of immunosuppression correlates with the UTI (Giessing, 2012; Arabi et al., 2021).

#### 2.3.9 Pregnancy

Pregnant women often experience urinary tract infections, especially pyelonephritis, that can be treated effectively in most cases. Women are more likely to acquire urinary tract infections due to alterations to the urinary system and immunologic changes during pregnancy. The ureter and renal calyces enlarge as a result of progesterone-related smooth muscle relaxation and ureteral compression from the gravid uterus, which are both physiological alterations of the urinary tract. Vesicoureteral reflux can be observed, and frequent urination is also caused due to decreased bladder capacity. Preterm labor, low birth weight, or systemic infection in the mother may be caused by pregnancy-related adaptive changes during UTI (Habak and Griggs, 2023).

## 3 Approaches for UTI detection

### 3.1 UTI biomarkers

**Figure 2:**
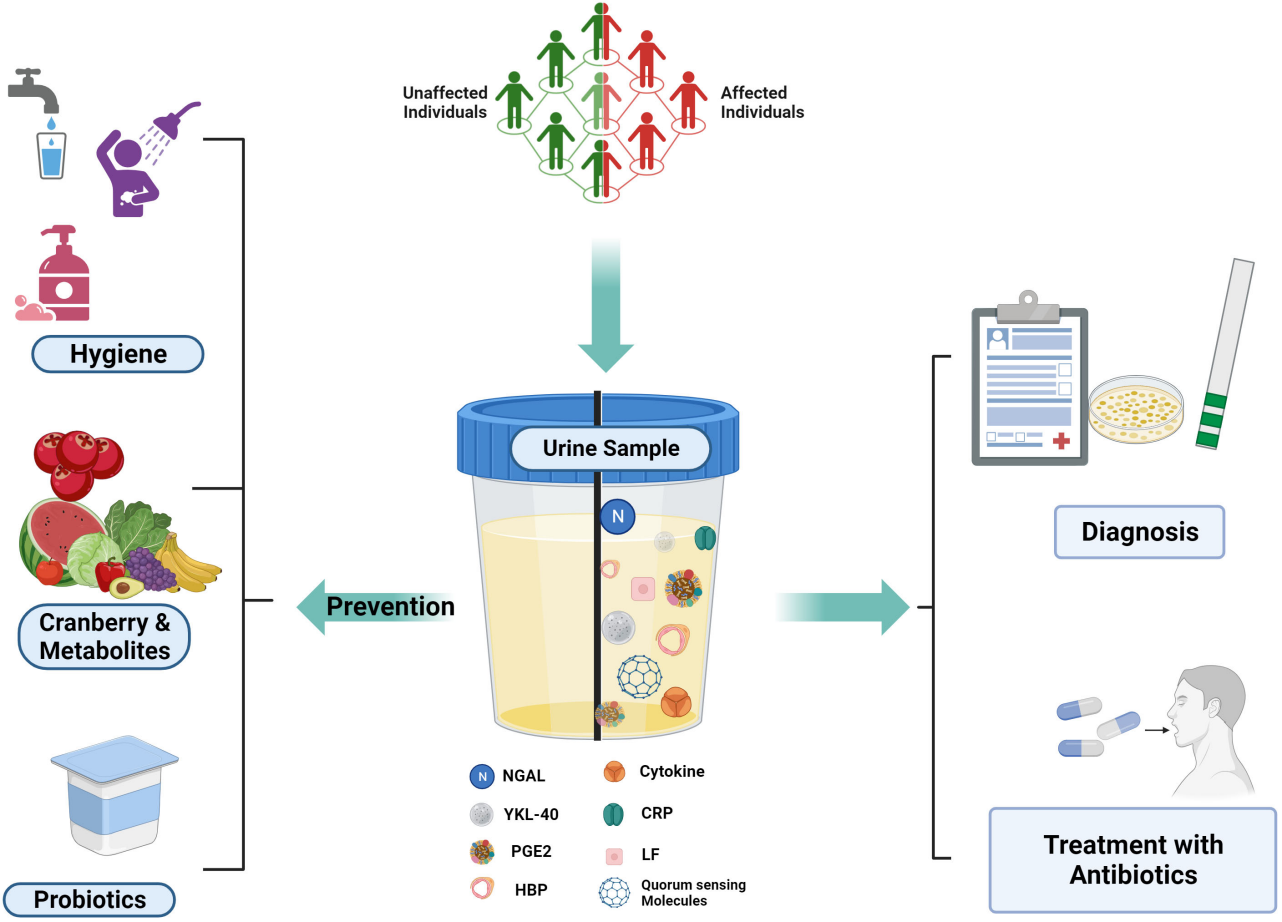
UTI Biomarkers and Prevention Strategies: Various host and pathogen markers for UTI Detection. *Created Using BioRender.com*.

A diagnostic biomarker validates the presence and severity of a disease and also helps to classify the disease, which helps in targeted diagnosis and treatment (Califf, 2018) (**Figure 2**).

#### 3.1.1 Host- based biomarkers

Lipocalin 2, or Neutrophil gelatinase-associated lipocalin (NGAL), is a 25 kDa acute phase iron carrier protein, highly expressed by human monocytes/macrophages and neutrophils and acts as a modulator in the innate immune response along with the oxidative stress response by distinct cell types and epithelial development. The transcription is initiated due to the lipopolysaccharides, and it hinders the growth of the bacteria by inhibiting the siderophores. Under normal circumstances, the levels of NGAL in plasma(pNGAL) and urine(uNGAL) are low, but they quickly rise in response to cell injury, particularly in cases of gram-negative UTIs indicating it as a potential biomarker for UTI. pNGAL and uNGAL are released during systemic inflammation genitourinary epithelium infection, respectively, and the levels are correlated with the duration of infection; they may also be utilized for monitoring as they rise within 12 h and peak in 3 days. (Ichino et al., 2009; Horváth et al., 2020; Moon et al., 2021; Yamamoto et al., 2023) Acute pyelonephritis and UTI in children with fever can be distinguished using uNGAL, according to a study by Moon et al. Compared to the non-UTI individuals, the individuals with UTI had a higher uNGAL level (Moon et al., 2021). Urbschat et al., in their study with 97 individuals, concluded that NGAL can be used as a biomarker for UTI (Urbschat et al., 2014). Yilmaz et al. evaluated the ideal cutoff level for uNGAL to consider it as a biomarker for UTIs. ELISA technique was employed to quantify the uNGAL in 29 healthy controls and 60 UTI patients. With a 20 ng/ml limit for uNGAL, the sensitivity and specificity obtained were 97% and 76%, respectively (Yilmaz et al., 2009). In contrast, Lubell et al. identified the threshold uNGAL in newborns and children as 39.1 ng/ml with a sensitivity and specificity of 97.1% and 95.6%respectively (Lubell et al., 2017). Jagadesan et al. also validated that NGAL at a cutoff level of 27ng/mL, NGAL can be used as a biomarker with 79.4% and 68.2% sensitivity and specificity, respectively (Jagadesan et al., 2019). The study by Price et al. demonstrated the potential of urine NGAL as a biomarker for adult female UTI diagnosis. They reported that the women with UTI had greater urine NGAL levels than the control out of the 50 UTI patients and 50 control subjects that were included. NGAL showed 100% specificity and 98% sensitivity with a threshold of 23.9 ng/mL (Price et al., 2017).

YKL-40, also known as cartilage glycoprotein-39 or chitinase-3-like-1 (CHI3L1), is a member of the chitinase-like protein family found in mammals and is expressed by many different types of cells, including chondrocytes, fibroblasts, some cancer cells, and some primary immune cells like neutrophils and macrophages.YKL-40 has been linked to several biological processes, including extracellular matrix remodelling, fibrosis, angiogenesis, and inflammation, even though its exact functions are unknown. YKL-40 is produced locally in inflammatory sites and may serve as a site-specific inflammatory marker. On the other hand, not much information about the connection between YKL-40 and UTI has been found (Kim et al., 2018; Mashaly et al., 2020). The study by Mashaly et al. compares the value of uYKL-40 to uNGAL in an attempt to evaluate it as a biomarker for UTI in children using ELISA. With a threshold value of 171.5 pg, 82% and 84% specificity and sensitivity, respectively, were obtained (Mashaly et al., 2020). Kim et al. used a sample size of 44 children with UTI and 35 children as controls to examine the association between fever and urinary tract infection in children. The concentration of YKL-40 in each sample was measured using ELISA. Urinary YKL-40/Cr levels were measured with a cut-off value of 125.23 pg mg^-1^ to detect UTIs. Of the nine children in the control group with pyuria, eight had levels less than 125.23 pg mg^-1^, and the single child in the UTI group without pyuria or positive nitrite had a level more than 125.23 pg mg^-1^ (Kim et al., 2018). The inflammation caused by cyclooxygenase-2 (COX-2) makes rUTI more susceptible and converts arachidonic acid to Prostaglandin E2 (PGE2), eliciting different responses such as angiogenesis, inflammation, pain perception, and cell proliferation. PGE2 is secreted at higher levels when COX-2 expression is induced (Ebrahimzadeh et al., 2021). The study by Ganguly et al. shows that PGE2 is a useful biomarker for quick, label-free UTI testing in terms of both diagnosis and prognosis. The novel electrofluidic capacitor-based biosensor based on affinity capture by monoclonal PGE2 antibody can be employed in less than five minutes for small-volume urine samples and provide high accuracy at home for managing UTIs (Ganguly et al., 2022).

Human neutrophil is secretory and azurophilic granules secrete a heparin-binding protein (HBP), commonly known as azurocidin or cationic antimicrobial protein of 37 kDa (CAP37) causes vascular leakage, development of oedema, act as a chemoattractant and has a wide range of antibacterial activity. HBP is released when neutrophilic β2-integrins ligate resulting in vascular leakage. Research has demonstrated that it is elevated. HBP levels in plasma are present in patients with severe sepsis before the onset of hypotension (El-Refaey et al., 2020; Linder et al., 2010). The Kjolvmark et al. study showed that urine levels of heparin-binding protein produced from neutrophils can serve as a signal for pediatric UTIs with a sensitivity and specificity of 93.3% and 93.3%, respectively at a cut-off level of 32 ng mL^-1^ (Kjölvmark et al., 2012). Rafaey et al. concluded that urinary heparin-binding protein could be utilized as a reliable biomarker for the identification of UTI using ELISA in infants, and the sensitivity and specificity were confirmed as 72.2% and 81.2%, respectively, at a threshold level of 650 pg mL^-1^ (El-Refaey et al., 2020). Kjolmark et al. assessed adult U-HBP as a UTI marker and compared it to IL-6 urine levels and the dipstick test in two distinct healthcare settings. As a UTI marker, HBP in urine has a sensitivity of 89.2% and a specificity of 89.8%, respectively, and was superior at differentiating between cystitis and pyelonephritis (Kjölvmark et al., 2014).

Small soluble peptides called cytokines are produced by macrophages, glomerular endothelial cells, and intestinal epithelial cells in the presence of uropathogenic bacteria causing inflammatory reactions. Mononuclear phagocytes release IL-1, which comes in two forms and serves as the initial cytokine in the antigen recognition immunological cascade accounting for acute-phase reactions and fever development. Multifunctional cytokine IL-6 controls several bodily processes, including inflammation, organ development, and the acute phase response. Hepatocytes, podocytes, T-helper cells, neutrophils, and macrophages all express the IL-6 receptor. TNF-α, IL-1, and IL-2 cause macrophages to release the IL-8, which attracts the neutrophils. IL-6 and IL-8 are present in trace amounts in a healthy individual and are assumed to be biomarkers of urinary tract infections and to be indicative of the infection site (Al Rushood et al., 2020; Gedikbaşı et al., 2020). A study by Gedikbaşı et al. demonstrated using ELISA that the amount of IL-1β in urine could serve as a reliable marker for urinary tract infections. With a threshold value of 6.11 pg mL^-1^, the sensitivity and specificity were 100% and 93.1%, respectively, IL-1β was expressed at a higher concentration in UTI patients compared to controls (Gedikbaşı et al., 2020). Using eighty serum samples and seventy-two urine samples taken from children with urinary tract infections, Abed et al. demonstrated that IL-8 is a good biomarker for urinary tract infection, while IL-6 is not. Their findings demonstrated that there was a considerable increase in IL-8 concentrations in both the serum and urine during UTI as compared to the control (abed et al., 2021).

In contrast, Abdelaal et al. demonstrated IL-6 as a UTI biomarker. The results of the study showed that patients with UTIs had considerably higher urinary IL-6 levels than the control group. The optimal cut-off value was 17 pg mL^-1^, with a sensitivity, specificity, and diagnostic accuracy of 94.4%, 92.1%, and 92.8%, respectively. Furthermore, they showed that urine IL-6 levels were positively correlated with leukocyte count, fever, CRP, and that urinary IL-6 levels were significantly higher in instances of acute pyelonephritis APN compared to milder UTI cases (Abdelaal et al., 2019). According to the study conducted by Mazaheri et al, IL-6 and IL-8 are both sensitive biomarkers of UTI that can distinguish between lower urinary tract infections and acute pyelonephritis (Mazaheri, 2021). Acute-phase inflammatory protein (CRP) produced by the hepatocytes under the influence of IL-6 is homopentamer and shows upregulated expression during inflammatory disorders, rheumatoid arthritis, and cardiovascular illnesses. CRP levels are 0.3–0.6 mg dL^-1^ in healthy people, and due to the infection, the serum concentrations surpass 5 mg L^-1^ within approximately 6 h and peak at 48 h, which has a half-life of roughly 19 h (Agrawal et al., 2013; Narayan Swamy et al., 2022; Tang and Zhou, 2022).

The function of blood C-reactive protein levels in upper and lower urinary tract infections in adult patients was investigated by Agarwal et al. They discovered that the upper urinary tract infection cases had a mean C-reactive protein value of 127.33 mg L^-1^, which is statistically substantially higher than the control (Mamatha, 2020). The majority of upper UTI patients diagnosed with acute pyelonephritis had CRP values >100 mg L^-1^, and lower UTI patients diagnosed with cystitis had CRP levels between 3 and 50 mg L^-1^, according to a study by Narayan Swamy et al (Narayan Swamy et al., 2022).

Lactoferrin, belonging to the transferrin family and a component of the innate immune system, is a single polypeptide chain glycoprotein that binds iron and has a molecular weight of about 78 kDa. Initially isolated from human breast milk and later it was discovered in bodily fluids such as saliva, tears, bile, pancreatic juice, and intestinal fluid. Being the initial line of defence against pathogens that enter through mucosal tissues, it is crucial in the identification of several clinical conditions, including Parkinson, inflammatory bowel disease (IBD), Alzheimer’s, and UTI (Fatah et al., 2016; Naseri et al., 2021).

The LF concentrations in the urine of 88 patient samples and 121 normal samples were measured using an ELISA in a study by Arao et al. The lactoferrin concentration in the UTI samples and healthy individuals was 3,300.0 ± 646.3 ng mL^-1^ and 30.4 ± 2.7 ng mL^-1^ respectively. With the threshold of 200 ng mL^-1^, the efficacy of the immunochromatography was measured and the sensitivity, specificity, positive, negative predictive values were found to be 93.3, 89.3, 86.2, and 94.9%, respectively (Arao et al., 1999). In a study by Fatah et al., the urine samples of the control had a concentration of 670 ± 319 ng mL^-1^ while it was 1387 ± 509 ng/ml during the infection and 885 ± 268 ng mL^-1^ after two months indicating higher concentrations during the infection and the significantly lower concentrations following two months (Fatah et al., 2016).

To detect lactoferrin, Naseri et al. developed an electrochemical biosensor with a broad linear range of 10 to 1300 ng mL^-1^, a LOD of 0.9 ng mL^-1^, high selectivity, and reproducibility (Naseri et al., 2021).

Numerous disorders (chronic, metabolic, or malignant disease, recurrent infections, etc.) constantly affect the immune system and can have an impact on biomarker expression for UTI. Urine testing for volatile organic chemicals or bacterial metabolites should be preferred (Horváth et al., 2020). Trimethylamine (TMA), acetate, and xanthine oxidase (XO) were shown to be potential markers in the review by Karlsen et al. Trimethyl quaternary amines (N(CH_3_)_3_)) are dietary sources of a volatile tertiary amine with a “shy odour” absorbed by the gut bacteria through the stomach walls and transferred to the liver, where it is converted by the avin-containing monooxygenase (FMO) enzyme into the odourless trimethylamine-N-oxide (TMAO), finally expelled through urine. In healthy individuals, TMA is nearly entirely transformed into TMAO, with a urinary TMAO excretion rate of up to 60 mg per day (Karlsen and Dong, 2015). The study by Lam et al. revealed that trimethylamine, a human-microbial marker associated with *E. coli*, established a threshold of 0.0117mmol with 97.0% specificity and 66.7% sensitivity (Lam et al., 2015). At physiological pH, acetic acid mostly occurs as the conjugate carboxylate base acetate with a pKa of 4.76, and the concentrations are high in the majority of the infections. Gupta et al. identified acetate, lactate, succinate, and formate as markers capable of discriminating between UTI patients and healthy controls with high sensitivity and specificity.

#### 3.1.2 Pathogen-based biomarkers

Small chemicals termed autoinducers diffuse into the environment, accumulate during the growth of the microbial population, and reach a certain threshold to mediate the cell-to-cell signalling system known as quorum sensing (QS) and attach to cytoplasmic or membrane-bound transcription factors to initiate gene expression and release additional signalling molecules. Clinical professionals can diagnose a disease if these signalling molecules are quickly detected and help curb the spread of antibiotic-resistant bacteria (Stapleton et al., 2011; Miller and Gilmore, 2020) (**Table 3**). Low molecular weight acyl-homoserine lactones (HSLs) and autoinducing peptides are secreted either passively or actively by Gram-negative and Gram-positive bacteria, respectively. HSLs are neutral lipid molecules that have a lactone ring with variable side chains of carbon, indicating the hydrophobicity of the compound (Miller and Gilmore, 2020). Among *P. aeruginosa* uroisolates, there were variations in the types and quantities of AHL generated. In these strains, several AHLs, C4-HSL, C6-HSL, oxo-C6-HSL, C8-HSL, C10-HSL, and C12-HSL families were identified (R. Kumar et al., 2011). N-octanoyl homoserine lactone and N-3-dodecanoyl-l-homoserine lactone are produced by *K.pneumoniae* isolates from human tongues (Miller and Gilmore, 2020). Montagut et al. produced antibodies directed against 2-heptyl-4-quinolone (HHQ), a signalling molecule from *P.aeruginosa* PQS QS system that is crucial for the synthesis of virulence components and the creation of biofilms. The ELISA that was developed has a LOD of 0.34 ± 0.13 nM (Montagut et al., 2020). To detect the quorum-sensing signalling molecules of gram-negative bacteria, N-acyl-homoserine lactones (AHLs), Sahana et al. developed a PL-based biosensor using ZnO nanoparticles functionalized with cysteamine (S. Vasudevan et al., 2020).

**Table 3:** Various quorum sensing molecules used for diagnosis of different diseases.

S.No.	Probable QS system that can be used	Detection technique	Sample collection	LOD	Merits	References
1	Pyocyanin	Polyaniline/Au nanostructures modified ITO electrochemical sensor	Clinical samples	500 nM	• Fast • precise	(Elkhawaga et al., 2019)
		Silver nanorod array based SERS	Sputum samples	2.38 x 10^-8^ mol L^-1^	• Multiple specimen • rapid	(Wu et al., 2014)
		Gold coated zein film SERS	Drinking water	25 μM	• Biodegradable	(Jia et al., 2019)
		ELISA immunochemical method	Sputum samples.	0.60 ± 0.01 nM	• Robust • reproducible • Multiple samples	(Pastells et al., 2016)
		Carbon fibre electrochemical sensor	–	0.030 µM	• Reagent free • Rapid • inexpensive consumables • performed by non-specialists	(Sharp et al., 2010)
		3D redox capacitor electrochemical sensors	Sputum	50 nM	• High sensitivity	(Kang et al., 2021)
			Wound fluid	100 nM		
			Urine	5 nM		
2	HQNO	Immunochemical approach	Clinical samples	0.60 ± 0.13 nM	• Fast • precise	(Montagut et al., 2022)
3	2-Heptyl-4-quinolone	Elisa immunochemical method	Sputum specimens	0.34 ± 0.13 nM	• Robust • Reproducible • specific	(Montagut et al., 2020)
4	4-hydroxy-2-heptylquinoline	Electrochemical method	Sputum	1.2 μM	• Low cost • easy to use instrumentation	(Burgoyne et al., 2020)
5	2-heptyl-3,4-dihydroxyquinoline	Electrochemical method	Sputum	1.1 μM	• Low cost • easy to use instrumentation	(Burgoyne et al., 2020)
6	2-heptyl-3-hydroxy-4-quinolone (PQS), and	Boron-doped diamond based electroanalysis	Sputum samples	0.15 μM	• Wide potential range • high current density • electrochemical stability • low background current • high resistance to fouling	(Buzid et al., 2016)
	2-heptyl-4-hydroxyquinoline (HHQ)			0.62 μM		
	pyocyanin			1.25 μM		
7	Quinolone	Elisa immunochemical approach	Sputum samples	0.36 ± 0.14 nM	• Robust • accurate	(Montagut et al., 2021)
8	1-hydroxyphenazine	ELISA immunochemical method	Sputum samples.	0.60 ± 0.01 nM	• Robust • Reproducible • Multiple sample detection	(Pastells et al., 2016)
9	3-oxo-C12-HSL	DNA-encoded biosensor	Sputum samples	1.56 nM	• Rapid • low-cost detection assays	(Wen et al., 2017)
10	AHL	Genetically engineered whole-cell sensing systems	Saliva samples	–	• minimal or no sample preparation • provide information regarding the bioavailability of the analyte.	(Kumari et al., 2008)
11	N-(3-oxo)-dodecanoyl-L-homoserine lactone	Electrochemical biosensor	Spiked saliva	2 pM	• High reproducibility • minimal sample preparation • rapid	(Baldrich et al., 2011)

### 3.2 Diagnostic methods for UTIs

**Figure 3:**
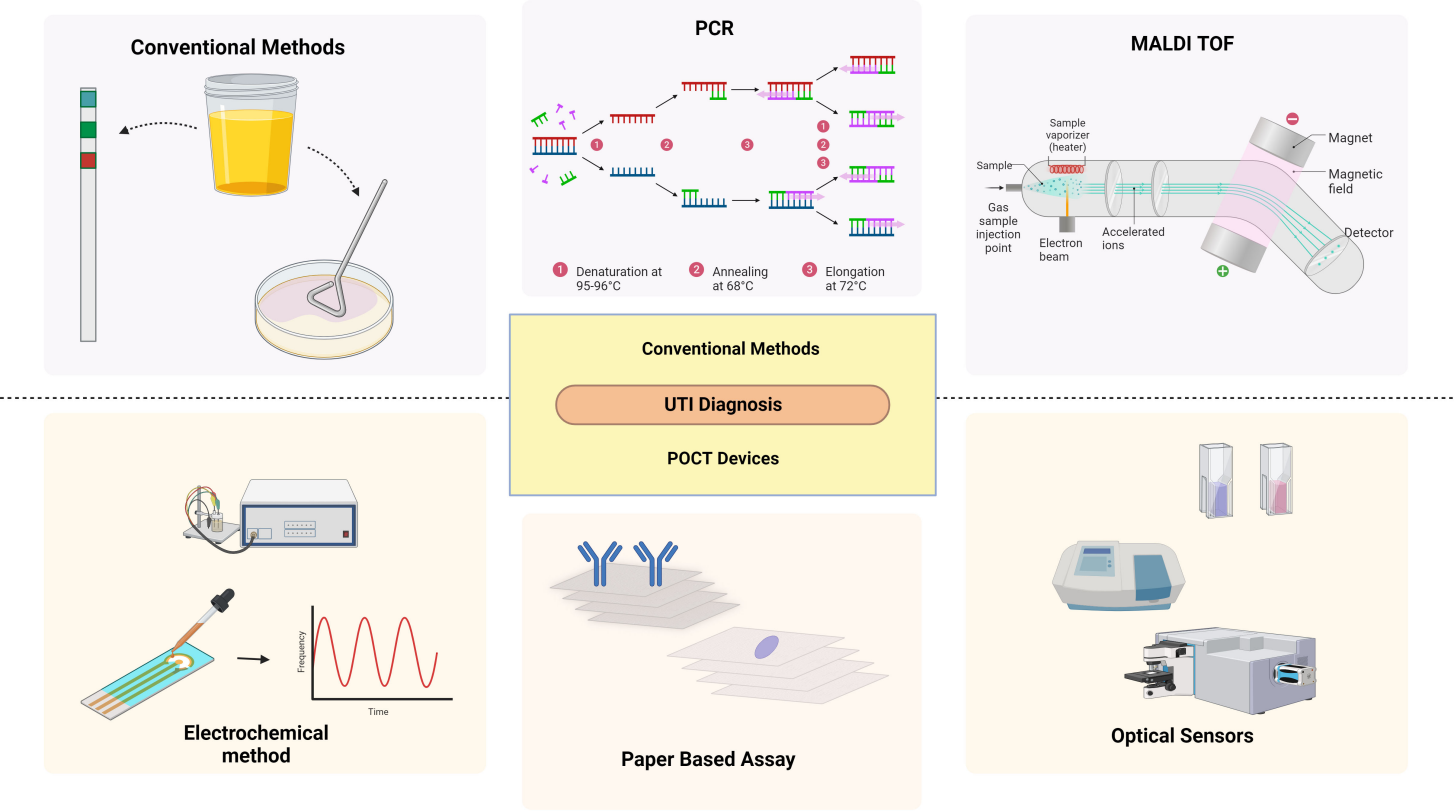
Diagnostic methods of UTI: Conventional and emerging methods. *Created Using BioRender.com*.

The term “diagnosis” has been defined as a classification scheme used by the medical community to group specific diseases that are pathological that aid in establishing a social order that identifies and diagnoses the disease promptly in healthcare and the pharmaceutical sector, suggesting the best course of treatment, and forecasting the results (Jutel, 2009; Chua et al., 2011; Balogh et al., 2015; Kumar et al., 2016). Even a slight delay enhances the risk of morbidity and mortality, necessitating early treatment that depends on rapid detection. (Arinzon et al., 2009; Fazly Bazzaz et al., 2021) The ideal test should be affordable, rapid, and accurate in the diagnosis of high-risk individuals. However, the diagnosis of UTI is challenging as the signs and symptoms can overlap with the risk factors (Mambatta et al., 2015) (**Figure 3**).

#### 3.2.1 Urinalysis

**Figure 4:**
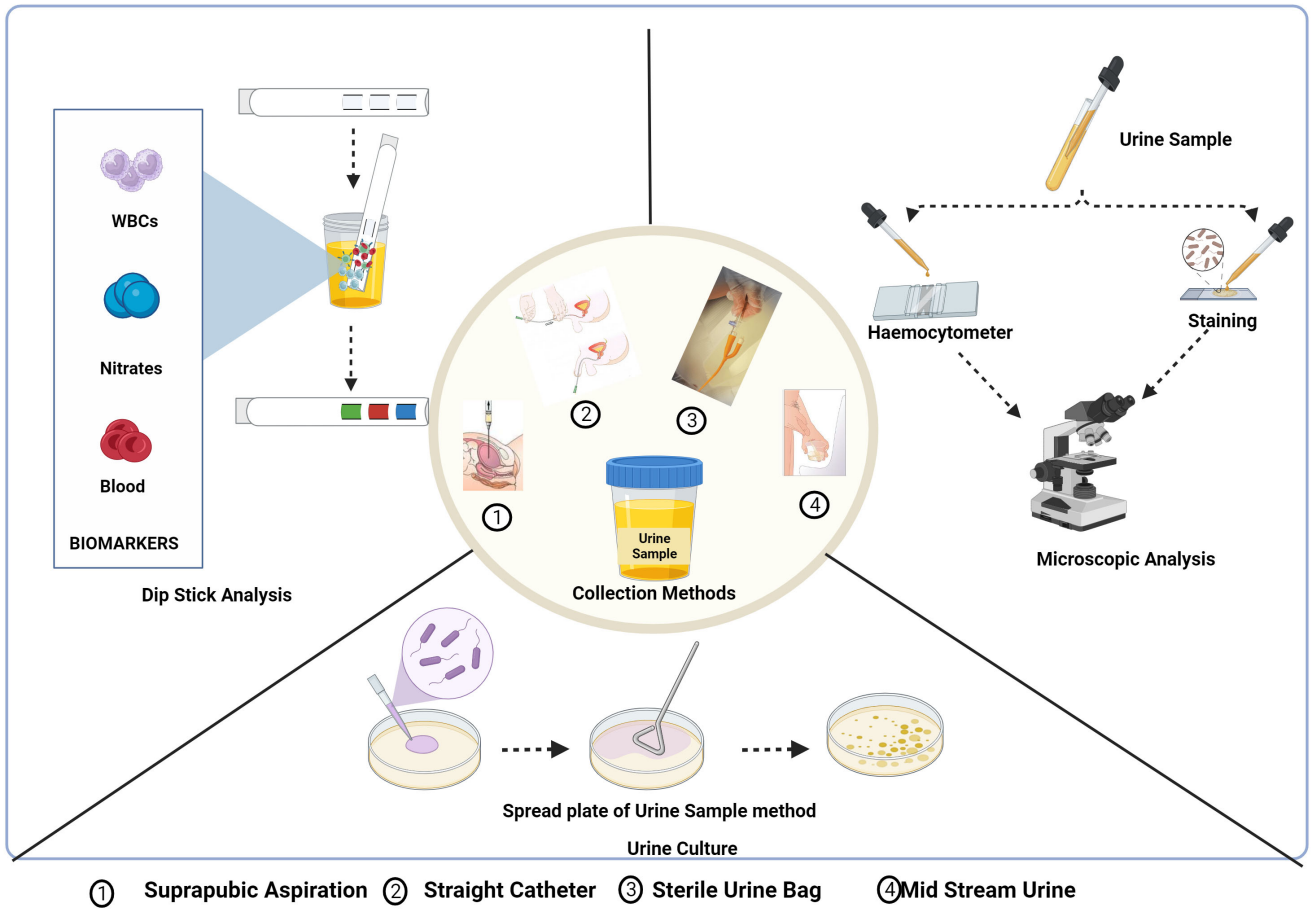
Urine collection methods and conventional diagnostic methods of UTI: Dipstick analysis, Microscopic analysis and urine culture test. *Created Using BioRender.com*.

An easy-to-use, quick, and cheap screening technique is urine analysis, which includes physical, chemical, and microscopic investigation (Mambatta et al., 2015) (**Figure 4**).

##### 3.2.1.1 Dipstick analysis

Dipstick urinalysis is the first step in urine testing that checks the presence of leukocyte esterase (LE), nitrite, and red blood cells based on symptoms and other characteristics shown by the patient (Chu and Lowder, 2018). Leukocytes, which are elevated in urine during UTI, express LE with a threshold limit for the detection of 5-15 WBC/high-power fields. The LE in the sample interacts and hydrolyses the ester substrates in the dipstick, turning it blue (Mambatta et al., 2015; Chu and Lowder, 2018). The presence of nitrite in urine is typically undetectable, but if the bladder holds the dietary nitrates in the urine for more than four hours, bacteria get sufficient time to reduce them to nitrite and the threshold value being 0.1 g/ml for the test to be positive indicated by a colour change in the strip (Andriole, 1987; Mambatta et al., 2015). The hematuria dipstick test is a screening test for UTI indicated by a colour change for a positive result due to the oxidation of a test-strip reagent. However, the microscopic analysis needs to be conducted for verification (Mambatta et al., 2015).

The purpose of a study by Mambatta et al. was to assess the sensitivity of urine dipstick analysis utilizing the Multistix 10 SG (Siemens) and Clinitek Advantus Analyzer. The sensitivity of nitrite, leukocyte esterase, and blood alone was found to be 23.31%, 48.5%, and 63.94%, respectively. When nitrite, leukocytes, and blood were taken into account collectively, the sensitivity turned out to be at its highest. They concluded that the nitrite test and LE test had low sensitivity and could not rule out UTIs in patients, and a urine culture needs to be performed for suspected patients with UTIs as the results of the Dipstick are unreliable in a few cases (Mambatta et al., 2015). Nitrite and red blood cell testing yield the results after one minute, and LE needs two minutes for the outcome. The interpretation of a dipstick urinalysis might be affected by several conditions.

##### 3.2.1.2 Microscopic examination

Nephrologists frequently use the diagnostic technique of urinary microscopy by direct or indirect examination of the centrifuged or uncentrifuged samples (Fogazzi et al., 1998). The procedure for the staining of an uncentrifuged sample is simple in which a small amount of urine is placed on a glass microscope slide, let to air dry, stained with gram stain, and then studied under a microscope (Wilson and Gaido, 2004). To diagnose UTIs brought by *bacilli* spp., including *cocci*, the study by Hiraoka et al. evaluated the value of microscopic examination of 172 unspun urine samples using a disposable slide with counting chambers. The approach indicated bacteriuria with 94% and 88% accuracy, respectively. Pyuria had a 79% and 71% sensitivity and specificity, respectively (Hiraoka et al., 1993). Similar research revealed that the sensitivity and specificity for 89 urine samples were 91 and 98%, respectively. Both studies stated that the microscopic examination of urine on disposable counting chambers was quick and simple to do without the need to set up or clean the chambers—reliable, affordable, and time-efficient (Hiraoka et al., 1995).

An alternative quick test for the intended purpose that is often used in Danish general practice is point of care (POC) microscopy. The clinician may determine the number of bacteria seen per field of vision, ascertain the morphology of the organisms (rods or cocci), and describe their type of motility (non-motility, polar or nonpolar motility) using POC microscopy using either a conventional light microscope or a phase-contrast microscope. The benefit of phase-contrast microscopy is that the specimen does not need to be stained. To prevent overdiagnosis, immersion oil and phase contrast microscopy, which showed high clinical accuracy and specificity, may be helpful as a follow-up test for urine dipstick (Beyer et al., 2019). Pyuria can be identified and quantified microscopically utilizing the urinary leukocyte excretion rate, counting leukocytes either by haemocytometer or by Gram staining or in the centrifuged sample. The ability to see leukocytes, leukocyte casts, and other cellular components up close is a benefit of urine microscopy. Leukocytes quickly degrade in urine that is not fresh, or that has not been properly preserved, which is a drawback of urine microscopy. Urinary leukocyte excretion rates in patients with symptomatic UTIs are 400,000 leukocytes/h (Wilson and Gaido, 2004).

##### 3.2.1.3 Urine culture and sensitivity

For urine culture, suprapubic aspiration, straight catheter technique, and mid-stream catch are the different collection methods that are influenced by the comfort of the patient, the capacity to void, and lowering the risk of iatrogenic infection. The most effective technique to prevent specimen contamination is to collect samples by suprapubical method. With the use of ultrasonography, a needle attached to a syringe is introduced through the lower abdomen into the bladder to collect the urine sample (Karacan et al., 2010). This procedure is rarely used as it is invasive, uncomfortable for the patient, and resources are utilized inappropriately. The next most suitable method is urine collection using a single catheter (straight catheter approach). However, this approach is rarely performed and only when necessary due to the labour intensiveness and probable risk of introducing bacteria into the bladder, which could potentially result in a UTI. A clean-catch midstream technique, which is neither intrusive nor painful, is the most typical way to obtain a urine sample for urine culture. The possible drawback of this method is that the sample can get contaminated with the commensal residing in the distal urethra. So, collecting midstream urine is recommended (Wilson and Gaido, 2004; Sinawe and Casadesus, 2023).

The test results are based on the urine collection method as well as its processing. The test requisition slip should include the date, time, and method of specimen collection, patient demographic information, and details regarding antimicrobial therapy, anatomic abnormalities, or indwelling urinary catheters. The Urine sample needs to be tested within 2 h of collection or should be refrigerated or preserved using a boric acid solution and is generally cultured in Blood agar, and MacConkey’s agar media is incubated at 37°C for 24-48 h. Patient-related factors, such as the use of antibiotics or diuretics, reduce the presence of pathogens, leading to false results. Additionally, improper collection or handling of the specimen also affects the test results (Wilson and Gaido, 2004; Sinawe and Casadesus, 2023).

Suprapubic aspiration had the lowest contamination rate, while sterile urine bags had the highest, according to a study done to assess the reliability of the four urine sample collection techniques among children suspected of having urinary tract infections. Urine culture costs increased due to the necessity to repeat procedures on contaminated specimens (Karacan et al., 2010). In another study, the non-invasive method, the clean-catch urine stimulation approach was employed to collect urine from infants younger than three years of age for the diagnosis of UTI. Urinary catheterization has a reduced but comparable contamination rate to clean-catch urine stimulation (Herreros et al., 2021).

#### 3.2.2 Imaging studies

Imaging can help make a diagnosis of UTI in newborns and infants as it is crucial when clinical and laboratory results are ambiguous or when atypical or vague clinical symptoms are present. The abnormalities elevate the risk for challenging treatment and long-term complications. Additionally, imaging aids in the detection of serious conditions, such as lobar nephronia, granulomatous pyelonephritis, pyonephrosis, necrosis/necrotizing pyelonephritis, and track the progression of the deformity in children who have had severe UTIs or malformations of the urinary system that are related to UTIs (Bjerklund Johansen, 2004; Riccabona, 2016). The most frequently used technique, named ultrasonography, is a non-invasive, adaptable, and affordable treatment that uses high-frequency sound waves to acquire real-time images of the area being scanned and allows the detection of urinary tract dilations and irregularities. Ultrasonography is prone to the appearance of artefacts caused by abnormal interactions between sound waves and tissues or air-filled cavities, which result in inaccurate reconstructions of their anatomical structures because it relies on the creation of images from rapid analysis and interpretation of data acquired by a transducer. The majority of these distortions are caused by abnormal interactions between sound waves and tissues or air-filled cavities, which result in inaccurate reconstructions of their anatomical structures.

#### 3.2.3 Molecular diagnostic methods

##### 3.2.3.1 Polymerase chain reaction

Molecular diagnostic techniques based on Polymerase Chain Reaction (PCR) are extensively employed in clinical laboratories and physician offices globally, leading to better medical treatment and patient outcomes for a variety of illnesses (Kelly, 2023). Wojno et al. assessed the use of multiplex PCR-based molecular testing to identify bacterial infections in 582 patients who exhibited symptoms. In 74% of cases, there was agreement between PCR and culture; in 34% of cases, both were positive, and in 40% of cases, both were negative. But in 26% of cases, there was a discrepancy between PCR and culture: in 22% of cases, PCR was positive, but culture was negative, and in 4% of cases, PCR was positive but culture was negative. They concluded that urine culture and multiplex PCR are equally effective at identifying and detecting bacteria (Wojno et al., 2020).

By employing 82 urine samples and a commercial real-time PCR blood pathogen test (SeptiFast®), Lehmann et al. assessed the viability of qualitative urine pathogen detection and compared the results with dipslide and microbiological culture. They discovered that the results of SeptiFast® pathogen identifications were available 43 h prior to that of the culture results, and 67 samples were dip slide culture positive while 61 samples tested positive for SeptiFast® indicating the test has a sensitivity and specificity of 0.82 and 0.60, respectively, for identifying infections (Lehmann et al., 2011).

The study by Hansen et al. demonstrated a real-time PCR technique for UTI diagnosis and uropathogen identification in 330 urine samples had a sensitivity and specificity of 97% and 80%, respectively. They concluded that this method could differentiate between bacteriuria and the absence of bacteriuria, identify the uropathogen implicated within 4 h of sampling, enable appropriate medication decisions to be made the same day, and significantly reduce the need for subsequent urine cultures (Hansen et al., 2013).

##### 3.2.3.2 Mass spectroscopy

For the past few decades, mass spectrometry (MS) -based techniques—particularly matrix-assisted laser desorption ionization time-of-flight mass spectrometry, or MALDI-TOF MS—have been effectively applied to routine clinical pathogen characterisation, including UTIs (Svetličić et al., 2022). Mass spectrometry involves ionizing chemical compounds into charged molecules by the addition or removal of one or more protons and measuring the mass-to-charge ratio (m/z) (Singhal et al., 2015). Ferreira et al. verified the MALDI-TOF using 206 samples by initially centrifuging the material at a low rpm and then at a higher rpm to exclude leukocytes and collect bacteria, respectively. In 94.2%, MALDI-TOF MS detected this microbe directly from the urine sample, indicating direct bacterial identification is possible in a short amount of time and with a high degree of accuracy, particularly when high concentrations of Gram-negative bacteria are present (Ferreira et al., 2010). Liu et al. showed that MALDI TOF can reduce the identification time (minimum 0.5 h) and AST (minimum 4 h) of the primary pathogens of UTI to 5–10 h. This significantly reduces inspection time and significantly aids in the timely diagnosis and treatment of UTI patients (Liu et al., 2022). The findings of a study by Oros et al. highlighted the critical role that sample preparation and storage conditions play in influencing the dependability of MS data analysis, and they suggest that it has great potential as a dependable high-throughput tool for microbial pathogen identification in human urine samples (Oros et al., 2020). Traditional methods for UTI diagnosis have their limitations, as shown in **Table 4**. Recognizing the importance of rapid and accurate diagnosis, new methods are emerging that aid in improving patient outcomes.

**Table 4:** Conventional Techniques and demerits.

Sl.No.	Method Of Detection	Probable Biomarker	Demerits of the Detection Method	References
1	Dip stick analysis	Nitrate	False negative results due to • diluted sample • presence of *Streptococcus* spp.	(Chu and Lowder, 2018)
		Leukocyte esterase	False negative results due to • Contamination of Urine sample • Consumption of Antibiotics • technical error	(Wilson and Gaido, 2004; Mambatta et al., 2015)
		RBCs	false-positive result due to • hemoglobinuria, • myoglobinuria, • menstrual blood, • concentrated urine, • vigorous exercise • presence of oxidising agents like hypochlorite and povidone, • exposure to air • the pH of the urine is less than 5.1	(Bacârea et al., 2021)
2	Microscopic Examination	Bacteria	Outcome affected by • proper patient guidance, • appropriate urine collection and handling, • adequate microscopic equipment	(Wilson and Gaido, 2004)
3	Urine Culture	Bacteria	• Contamination of urine sample, • Handling of Sample	(Wilson and Gaido, 2004)
4	Imaging Techniques		Lack of high quality images due to • patient’s characteristics, • morbid obesity, • the presence of gas in the intestine, • lesions, and abnormalities	(Bjerklund Johansen, 2004; Riccabona, 2016)
5	PCR	Bacteria	• Preparation of Primers • Expensive • Skilled Personnel required	Yang and Rothman, 2004)
6	MALDI -TOF	Bacteria	Poor species discrimination due to • inherent similarities between organisms • misidentification due to a small number of spectra in the database	(Rychert, 2019)

### 3.3 Emerging trends in UTI diagnosis

The term point of care testing (POCT) is used to describe tests that are carried out near the patient rather than at a central laboratory, which reduces the detection turnaround time (St-Louis, 2000; Luppa et al., 2011; Shaw, 2016). Point-of-care diagnostic devices are adopted by healthcare professionals, patients, and their families for their user-friendly features (Konwar and Borse, 2020). The POCT Device was first introduced in 1962 to rapidly analyse blood glucose levels, and later, in 1977, rapid pregnancy test kits were developed, which led to the development of a trend for personalised diagnostics. The use of POC diagnostics to cover a wide range of conditions promises benefits and helps improve the health status of a wide range of the population in a developing country like India, which is resource-constrained in terms of healthcare developments and advances (Clark and Lyons, 1962; Yetisen et al., 2013; Konwar and Borse, 2020). POC tests, which are quick, affordable, and suitable for remote locations, should replace the traditional expensive lab-based diagnostic approaches, and the devices need to follow the ASSURED criteria that include affordable, sensitive, specific, user-friendly, rapid and robust, equipment-free, and deliverable (Garcia et al., 2015; Konwar and Borse, 2020; Naseri et al., 2022) (**Table 5**).

**Table 5:** Commercially Available UTI Diagnosis POCT Devices.

Commercially available POCT Devices					
S.No.	POCT Device	Principle/working of the instrument	Method of Detection	Time taken	Reference
1	FLEXICULT SSI-Urinary Kit	Petridish is divided into 6 compartments in which 5 are of equal size for antibiotic susceptibility and 1 is larger for quantitative analysis. The urine sample is poured and the results are analysed the following day after incubation.	Culture based method	Approx. 24 hrs	(Blom et al., 2002)
2	Uricult Trio	It consists of a dip slide with CLED agar on one side and both colourless base agar incorporated with 8-hydroxyquinoline-b-D-glucuronide and [Fe(III)] as substrate and indicator molecule and MacConkey agar for the detection of the b-GCNDase enzyme on the other side. b-GCNDase splits the substrate into glucuronic acid and 8-hydroxyquinoline and the latter, in the presence of iron ions, produces an insoluble complex which, after incubation at 37°C for 24 h, dyes the colonies with a characteristic black colour. The base agar medium furthermore incorporates bile salts to inhibit the growth of gram-positive cocci.	Culture based method	Approx. 24 hrs	(Dalet and Segovia, 1995)
3	KAR® device	The device consists of the receiving and the dispensing part. The former consist of 9 different wells which contains the culture media, chromophore and the reagent. After the addition of the sample, the results are observed the after 24 hours with a colour change.	Culture based method	8 hours	(Jover-García et al., 2020)
4	Diaslide urine culture	The Diaslide is composed of two plastic sections and are filled with MacConkey agar and CLED. The inoculation and dilution streaking in both agars are made possible by dipping the V-shaped inoculator into the urine sample and pulling the sampler from the other end through the device. After the inoculation process, the sampler is disposed of and the case is placed in a tray for standing incubation. Without opening the device, growth is seen.	Culture based method	Approx. 24 hrs	(Rosenberg et al., 1992)
5	uriscreen	The basis of Uriscreen is the detection of the bacterial catalase enzyme. Urine is drawn into a test tube that has been filled with the powdered Uriscreen reagent and hydrogen peroxide is then added, and the mixture is gently shaken for five seconds. A successful outcome is demonstrated by the creation of enough foam within one to two minutes of the hydrogen peroxide addition to form a complete ring or layer on the surface.	Enzymatic method	2 minutes	(Waisman et al., 1999)
6	Dipstreak	Two different kinds of agar are affixed back to back to a plastic paddle in the DipStreak apparatus. The prong tips that are attached to the end of the paddle are dipped into the urine sample, that inoculate the agar. Colonies are isolated between the range 103 and 107 CFU/ml.	Culture based method	Approx. 24 hrs	(Scarparo et al., 2002)
7	Urisys 2400 automated urine analyser	The flow cell measures the specific gravity using refractometry.	urine analyser		(Chien et al., 2007)
8	Urisys 1100	The LED illuminates at three different wavelength and the detector measures the amount of light reflected by the test strip used for the computation of the results.	Colorimetry	1 min	(Harris and Fasolino, 2022)
9	Clinitek Status	The reflectance spectrophotometer, measures the colour and intensity of light reflected from the reagent area and generates the results.	Colorimetry	1 min	(Chien et al., 2007)
10	Aution Micro	Using the refractive index method, the specific gravity of the urine is measured and the values are adjusted based on the protein and glucose concentration.	Colorimetry	45 seconds	(Chien et al., 2007)
11	Urilyzer	The LEDs emit the light onto the test strips placed on the strip tray and then, the reflected light based on the concentration of the analyte is detected by the detector that provides the output.	Colorimetry	1 min	(Pernille et al., 2019)
12	MBS POCT	It uses mono-use, disposable reaction vials in which samples are inoculated and quantifies the catalytic activity of redox enzymes in the primary metabolic pathways of bacteria. The log of bacterial concentration is inversely correlated with the amount of time required to produce a colour change.	Enzymatic method	5.24 hours	(Arienzo et al., 2020)

#### 3.3.1 New approaches for point of care devices

##### 3.3.1.1 Paper-based assay

**Figure 5:**
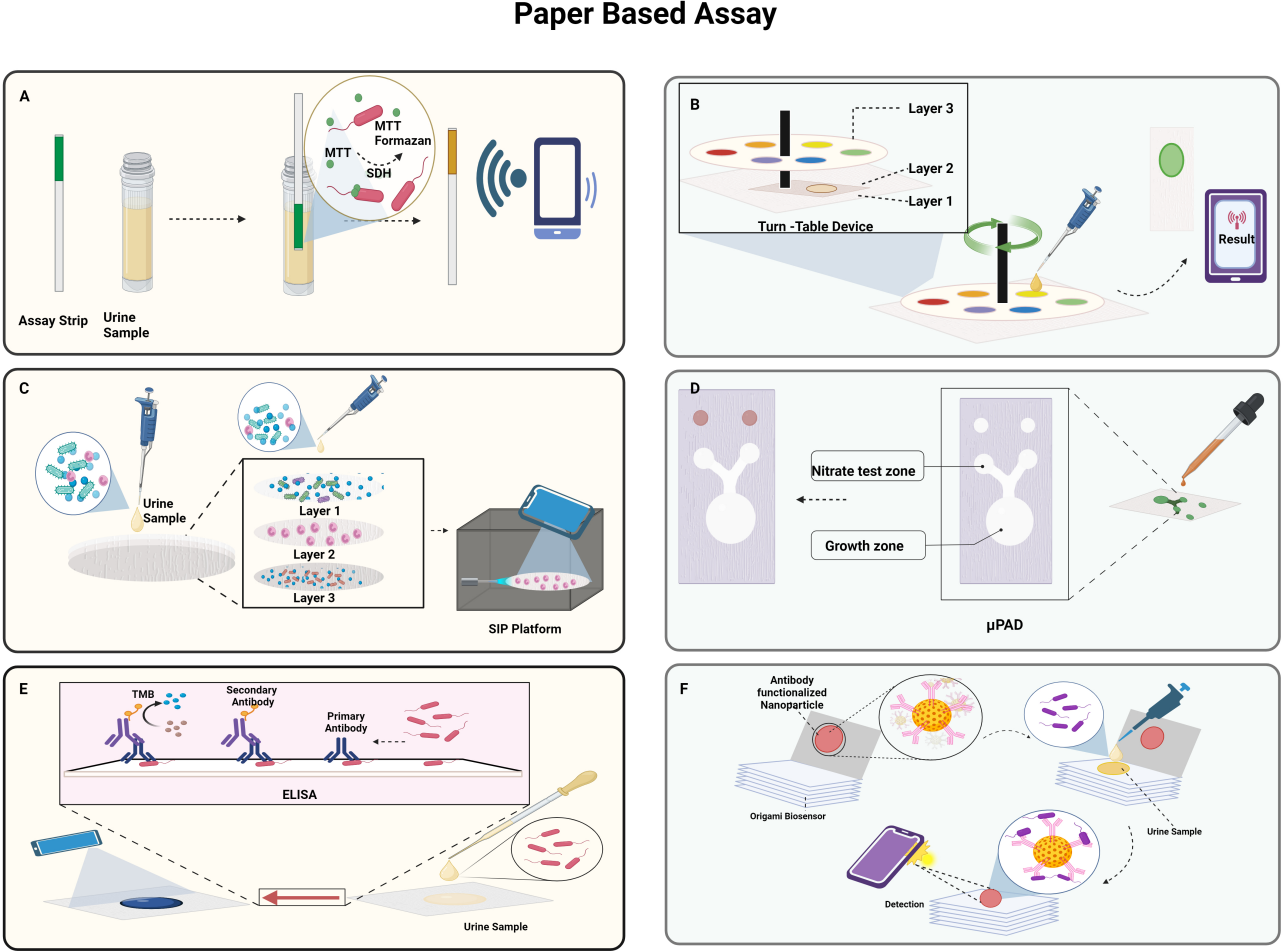
Paper-based approaches to detect UTI: **(A)**. Schematic representation of MTT-PMS test strips; **(B)**. Turn table paper-based device; **(C)**. Smartphone integrated paper (SIP)-based device; **(D)**. Paper-based analytical device (PAD) for detection of nitrate and *E.coli*; **(E)**. paper-based ELISA; **(F)**. Portable origami cellulose immunobiosensor. *Created Using BioRender.com*.

In the study conducted by Muljadi et al., the practicality of the MTT-PMS test strips was evaluated using *E.coli* as depicted in **Figure 5A**. The latter has a system of dehydrogenases capable of converting MTT to MTT-Formazan. The test strips were prepared by attaching the Whatman Fusion 5™ paper test pad to the MTT-PMS coated paper and then immersing it in a diagnostic reagent for five minutes, and storing it after drying it in the dark for 24 h. The assay was based on the colorimetric method, which involved dipping the strips for two minutes in the pretreated sample, letting it dry for twenty minutes, and recording the colorimetric data using a smartphone application, ColourPicker. They observed that the MTT-PMS strips could be used to differentiate the quantities of bacteria in a sample, and the correlation was calculated to be 98%. The strips might be employed as a quick and practical early alternative bacterium screening technique for a range of purposes (Muljadi et al., 2021). Wang et al. developed a turntable paper device consisting of three layers of paper, a wooden chopstick rotation axis in the centre, and an acrylic base at the bottom as depicted in **Figure 5B**. Blotting paper, Whatman® Grade 3 filter paper and Whatman® Grade 1 filter paper, respectively, comprised the first, second, and third layers. The filter paper in layer 2 in a rectangular shape is rotatable and was wax printed with a circular reactive zone. Six hydrophobic test zone sections were wax printed onto circular layer 3 and were fixed with pins. After loading a bacterial suspension onto the second layer of paper, it was given an hour to dry. Following the device assembly, reagents were gradually added to the third test zone, and the axis was adjusted to position the sample zone beneath the layer 3 reaction zone. Ultimately, after 2 minutes, the generated colorimetric reaction was captured on camera, and the images were analysed to determine the intensity of colour which correlated with the log scale of the bacterial cell concentrations (Y.-C. Wang et al., 2021).

A smartphone-integrated paper (SIP)- a device based on rapid on-site UTI screening- was proposed by Janev et al. as depicted in **Figure 5C**. A filtration system to separate the target cells WBCs from the heterogeneous components in the urine sample comprised of three layers of paper with various pore sizes. The sodium fluorescein dye in Layer 1 stains to the WBCs in the urine sample and is gathered in Layer 2 while the smaller cells are passed to Layer 3. Subsequently, the filter device is disassembled to acquire layer 2, and it is then positioned beneath a fluorescent microscope integrated with a smartphone. This microscope uses the rear lens of the smartphone to capture the fluorescence images of the WBCs, and the UTI is quantified by the processing of the image with a sensitivity and specificity of 96.67% and 100%, respectively. The use of smartphones enables to instantly share the collected data with the medical staff for real-time UTI patient monitoring and diagnosis and the whole detection process can be accomplished in less than ten minutes, which enables quick and precise UTI screening (Janev et al., 2023).

The study by Noiphung et al. was set out to develop a low-cost, portable paper-based analytical device (PAD) that quickly tests the presence of nitrite and simultaneously cultivates bacteria *in situ* as depicted in **Figure 5D**. A cotton sheet on top of Whatman No. 1 filter paper that had been wax-printed with a pattern to support the bacterial growth. The Griess reaction was utilized to sense nitrate, and X-GlcA, which was aliquoted into each PAD’s growth area, served as a substrate for the identification of bacteria. It was determined that the linear detection range of nitrate was 0–1.6 mg/dL. *E.coli* produces a β-glucuronidase enzyme that converts colourless X-GlcA substrate into blue colour. In 6 h, under ideal circumstances, the suggested apparatus could quantify bacterial concentrations in the range of 10^4^–10^7^ CFU/mL (Noiphung and Laiwattanapaisal, 2019).

A paper-based ELISA point-of-care diagnostic tool was developed by Shih et al. to quickly identify *E.coli* as depicted in **Figure 5E**. This involved adding the sample to the paper surface, letting it dry for an hour, and then blocking it with BSA. After 30 minutes each, horseradish peroxidase (HRP)-conjugated streptavidin and anti-*E. coli* biotin conjugate antibodies were added such that they conjugate with the sample. Following the addition of the substrate solution, the colorimetric findings were captured using a smartphone after two minutes. In just five h, the paper-based colorimetric platform could identify *E. coli* contamination. The average colour intensity on this platform is 0.1187 ± 0.002 for samples containing 10^5^ cells/mL and 0.01457 ± 0.003 for samples that are not contaminated (Shih et al., 2015). Adrover-Jaume et al. developed a portable origami cellulose immuno biosensor that can identify *E. coli*-caused UTIs at the bedside in less than seven minutes as depicted in **Figure 5F**. The device consists of a single piece of paper folded in an easy origami pattern and loaded with nanoparticles functionalized with antibodies. After the application of the urine sample in the paper in a reservoir containing polystyrene sulfonate (PSS), it is allowed to dry. The paper is then folded such that the nanoparticles are moved to the detection area and bind to the *E.coli*, causing coloured spots to appear on the paper strip due to the localized surface plasmon resonance (LSPR) of the gold nanoparticles at 531nm. The biospecific identification of the pathogen by the antibody is correlated with the pixel intensity of the spot measured using a smartphone app that operates in real time. They concluded that the assays exhibited high specificity and selectivity with other prevalent uropathogens as evidenced by the single false negative result they produced when tested using a panel of 57 patient urine samples (Adrover-Jaume et al., 2020).

##### 3.3.1.2 Electrochemical sensors

**Figure 6:**
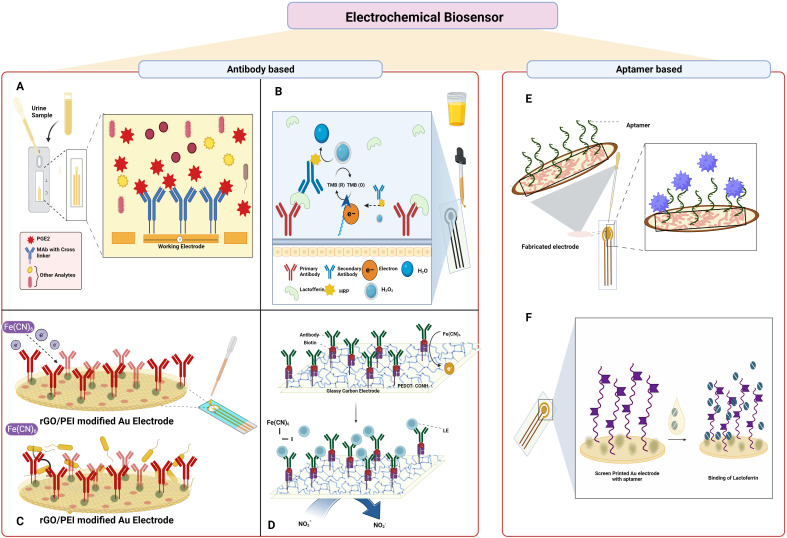
Electrochemical sensor-based approach to detect UTI: **(A)**. Lateral flow-based electrochemical biosensor for detection of urine PGE2; **(B)**. Electrochemical immunosensor for detection of Lactoferrin **(C)**. Electrochemical immunosensor for detection of UPEC; **(D)** electrochemical biosensor for detection of leukocyte esterase and nitrate; **(E)** Aptamer-based electrochemical sensor for detection of 3-O-C12-HSL; **(F)** electrochemical aptasensor for detection of lactoferrin. *Created Using BioRender.com*.

The study by Ganguly et al. showed the quantification of urine prostaglandin E2 (PGE2) for UTI diagnosis using a lateral flow-based electrochemical biosensor, as shown in **Figure 6A**. The affinity-based sensors were built by the deposition of the gold microelectrodes on the Cytiva Fusion 5 lateral flow membrane using a shadow mask in a cryo e-beam evaporator. The capture probe monoclonal PGE2 antibody was crosslinked to the gold electrode using DSP, and due to the interaction between the antibody and PGE2, the sensor detected the PGE2 using electrochemical impedance spectroscopy. The sensor predicts as UTI positive or negative in five minutes and offers a broad dynamic range of 100–4000 pg mL^-1^ (Ganguly et al., 2021).

The development of an electrochemical immunosensor using lactoferrin as the biomarker was reported, as shown in **Figure 6B**. A self-assembled monolayer (SAM) of alkanethiolate consisting of 6-mercapto-1-hexanol and 11-mercaptoundecanoic acid characterized with the help of electrochemical impedance spectroscopy was employed in an electrochemical biosensor array. Biotin was added once the sensor surface was activated which facilitated the binding of the streptavidin-attached antibody. The secondary antibody conjugated with HRP captures lactoferrin in the urine and the presence was detected with the help of amperometry measurements at -200 mV. The limit of detection determined through the analysis of 111 urine samples was found to be 145 pg mL^-1^ (Pan et al., 2010).

The fabrication of an immunosensor for sensitive and selective electrochemical detection of UPEC was described by Jijie et al. as shown in **Figure 6C**. Using the electrophoretic deposition approach, the gold electrodes were modified with thin active layers of reduced graphene oxide/polyethylenimine (rGO/PEI). Later, the electrode surface was again covalently modified with anti-fimbrial *E.coli* antibodies by amide bond formation that binds specifically to the *E.coli*. The decrease in the current due to the restriction of electron transfer from the redox probe potassium ferrocyanide to the modified electrode as a result of the immune complex formation served as the basis for the identification of *E.coli* and was measured using differential pulse voltammetry (DPV). The developed immunosensor had a detection limit of 10 CFU mL^-1^ and a linear range of 1 × 10^1^–1 × 10^4^ CFU mL^-1^ (Jijie et al., 2018).

An electrochemical biosensor was developed by Tseng et al. to detect leukocyte esterase and nitrate in clinical samples as shown in **Figure 6D**. The study was demonstrated by creating LE antibody conjugated to a modified glassy carbon electrode (GCE) with carboxylic acid-functionalized poly(3,4-ethylenedioxythiophene) (PEDOT-COOH), namely, LE antibody/Avidin/EDC-NHS/PEDOT-COOH/GCE. The presence of LE is indicated by the inhibition of the electron transfer by the [Fe(CN_6_)] and that of the nitrate by the DPV. The constructed electrode demonstrated good performance under optimal conditions, with a linear range of 9.1–131 µM and 0.2–590 μg L^−1^, and limits of detection for NIT and LE of 6.24 µM and 0.2 μg L−1, respectively. The outcomes from urine samples from thirteen patients showed 100% diagnostic sensitivity and 87.5% specificity when compared to the strip test (Tseng et al., 2023).

Capatina et al. developed an aptamer-based electrochemical sensor that could detect the 3-O-C12-HSL as shown in **Figure 6E**. The electrode surface was initially modified with gold nanoparticles, and then the C-SPE/AuNPs surface was immobilized with the aptamers, and later, the surface was blocked with MCH solution. After each modification step, the electrode surface was characterised using cyclic voltammetry, DPV, and electrochemical impedance spectroscopy using different redox probes. After the preparation of the electrode, the analyte 3-O-C12-HSL was added, and an electrochemical signal was determined. The presence of the analyte was also determined in spiked urine samples, spiked microbiological growth media, and microbiological cultures. The developed aptasensor had a LOD of 145 ng mL^−1^ (0.5 µM) and could detect the analyte between the range of 0.5–30 µM (Capatina et al., 2022).

Naseri et al. developed a label-free electrochemical multivalent aptasensor for the detection of human lactoferrin in urine samples as shown in **Figure 6F**. The materials gold, silver, and gold served as the working, reference, and counter electrode in the screen-printed gold electrodes, and the polynucleotide multivalent aptamer sequence was immobilized on the electrode. DPV was performed using 0.1 M acetate buffer solution as a blank to check the presence of human lactoferrin using a spiked buffer or artificial urine medium. The multivalent aptamer exhibited excellent sensing performances against human lactoferrin and had strong affinity and specificity with a broad linear range of 10 to 1300 ng/mL and a LOD of 0.9 ng/mL (Naseri et al., 2021).

##### 3.3.1.3 Optical sensors

**Figure 7:**
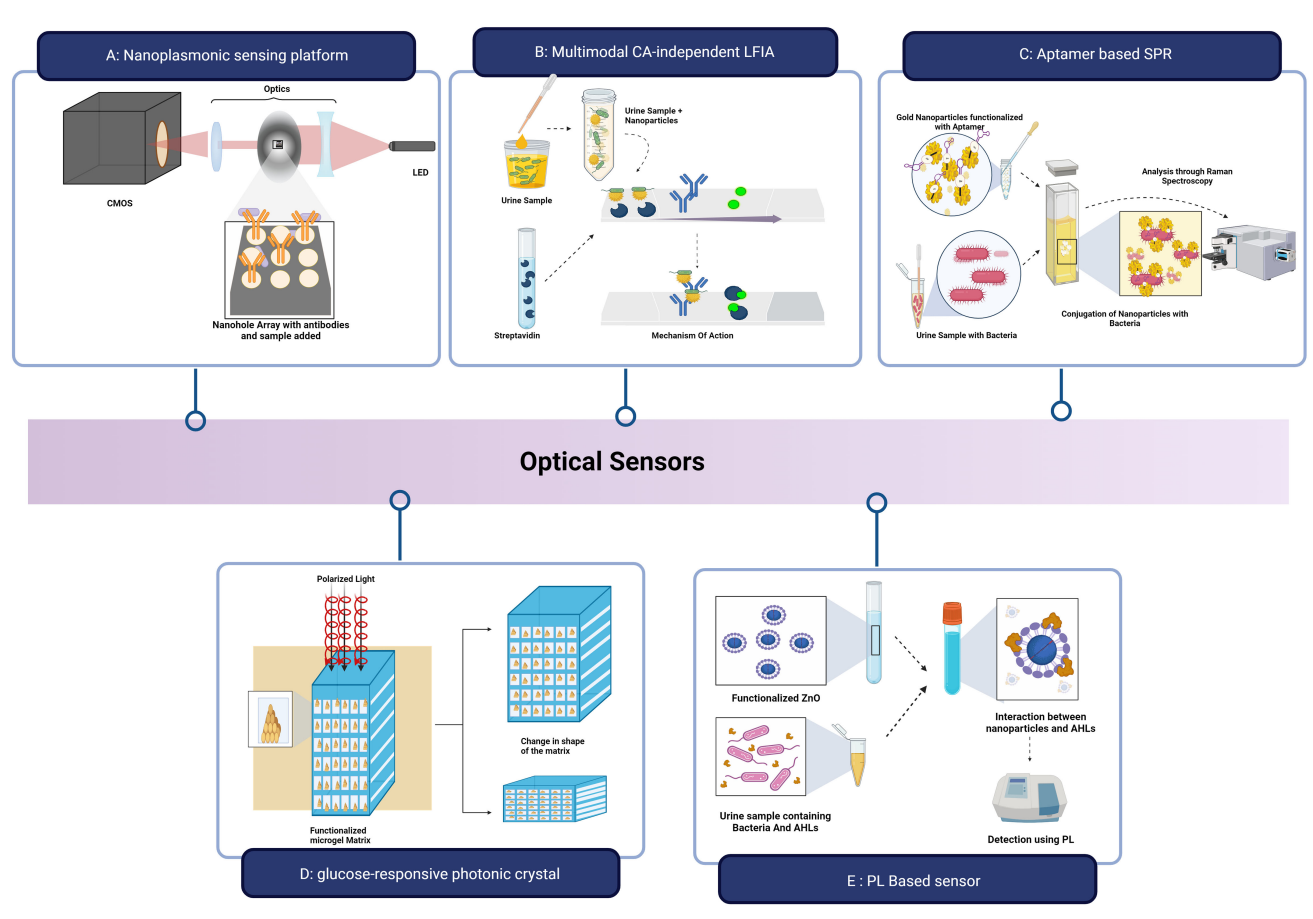
Optical sensors for UTI Detection: **(A)** Nanoplasmonic sensing platform for detection of UPEC; **(B)** Lateral Flow Assay for detection of *E.coli*; **(C)** Aptasensor to detect *K pneumoniae*; **(D)** Glucose-responsive photonic crystal integrated optical sensor; **(E)** Photoluminescence based optical biosensor. *Created Using BioRender.com*.

Gomez-Cruz et al. developed a label-free nanoplasmonic sensing platform to detect UPEC in real-time at concentrations below the physiological limit for UTI diagnosis as shown in **Figure 7A**. The sensing platform consisted of a metallic flow-through nanohole array-based sensor in conjunction with a Raspberry Pi interface, lens assembly, CMOS detector, and red LED light source. The light from the LED, after passing through the nanohole containing the UPEC antibody complex, causes the variation in the intensity of the transmitted light that is measured using surface plasmon resonance imaging. With a detection limit of 100 CFU mL^-1^, a resolution of approximately 10^−6^ RIU and a bulk sensitivity of 212 pixels per intensity unit (PIU)/refractive index unit (RIU) are observed (Gomez-Cruz et al., 2018).

Wu et al. described a multimodal CA-independent LFIA (MCI-LFIA) technique with adaptability and accuracy for the quick identification of bacterial UTIs as shown in **Figure 7B**. The Lateral Flow Assay employed *E.coli* as the model bacteria and AuNF − PMBA NMs (Gold nanoflower by p-mercaptophenylboronic acid nanomaterials) and AuNP-streptavidin nanoprobes as the test line (T-line) and control line (C-line) probes, respectively. The sample was initially incubated with AuNF − PMBA solution to obtain AuNF − PMBA − bacterium conjugates, and the conjugates, along with AuNP-streptavidin conjugates, were then applied to the test strip sample pad. Qualitative data was obtained by using high-accuracy visual observation to determine if the blue-green colour band of the T-line was present or absent within 45 minutes. The LOD for *E. coli* was 10^3^ CFU/mL in the colorimetric mode, 10^2^ CFU mL^-1^ in the Raman mode, and 10^2^ CFU Ml^-1^ in the photothermal mode (Wu et al., 2023). Using the shift in surface plasmon resonance of aptamer-modified AuNPs (Apt-AuNP), Deb et al. created an aptasensor to rapidly, precisely, and accurately identify *K. pneumoniae* (**Figure 7C**). Aptamers were immobilized on the gold surface to form the Apt-AuNP. The presence of the *K.pneumonia* bacteria in the urine sample causes the attachment of the Apt-AuNPs to their surface, and the UV–vis signal is intensified by producing a hyperchromic effect (Deb et al., 2023).

The ability of the glucose-responsive photonic crystal integrated optical sensor to measure glucose in urine for the diagnosis of UTI and glucosuria was demonstrated by Chowdhury et al. as shown in **Figure 7D**. Using incident electric polarized light, gold nanoparticles were chosen as the photonic crystal material and implanted into 36 blocks of a functionalized microgel matrix that was placed on top of a silicon substrate. A shift in the concentration of glucose resulted in wavelength shifting. The microgel swelled as the glucose concentration increased, increasing stack spacing and causing a rise in shift. On the other hand, as the concentration of glucose decreased, the microgel contracted. The sensor demonstrated enhanced sensitivity of less than 85.65 nm/mM at pH 7.4 and less than 110.60 nm/mM at pH 8.0 (Chowdhury and Zubair, 2022).

Vasudevan et al. developed a photoluminescence-based optical biosensor using cysteamine-capped Zinc Oxide (ZnO) nanoparticles that detects the AHL present in the sample. Prepared ZnO nanoparticles are functionalized with the linker molecule cysteamine as shown in **Figure 7E**. They can detect and bind to the quorum-sensing molecules, AHL, produced by the bacteria. The variations in the PL-based signals due to the surface defects allowed the detection of the analyte was validated using C4-HSL and 3-oxo-C12 HSL with a detection range of 10-120nM and a sensitivity of 97% (Vasudevan et al., 2020).

##### 3.3.1.4 Other Devices

For the diagnosis of UTIs, Michael et al. developed an instrument-free lab on a disc platform named diagnostic fidget spinner (Dx-FS). The device preloaded with the FAST solution was filled with a raw urine sample. The device is spun twice to concentrate the bacterial cells, followed by the removal of the FAST solution and then after the addition of the detecting solution. The colour change observed after 45 minutes was determined using the rapid colorimetric WST-8 assay that involved the transfer of electrons from living bacterial cells to the WST-8 by the electron mediator, which led to the visualization of the colour change of the formazan dye. The device allowed the on-site detection of urine samples from 39 individuals suspected of having a urinary tract infection in Tiruchirappalli, India, within 50 minutes using the naked eye (Michael et al., 2020).

The Micro Biological Survey (MBS) method was developed by Roma Tre University in Rome, Italy, as a colorimetric technique for the quantification of bacteria in various samples. It uses mono-use, disposable reaction vials in which samples are inoculated without any prior treatment and quantifies the catalytic activity of redox enzymes in the primary metabolic pathways of bacteria. The log of bacterial concentration is inversely correlated with the amount of time required to produce a colour change (Bottini, 2013). Arienzo et al. conducted a study to validate the MBS POCT. 1 ml urine sample was manually placed into a UBQ vial and was incubated in the MBS Multireader at 37°C and the bacteriuria is indicated by a colour change from blue to yellow. Positive results were indicated by a colour change that occurred within 5.24 h, whereas slower or no colour change occurred during the 24-h analytical timeframe. The MBS POCT urine analysis results were compared to the usual culture-based test results and the results showed 97% accuracy, 92% sensitivity, 100% specificity, 99% PPV, and 96% NPV within a 5-h analytical time threshold (Arienzo et al., 2020).

By using retrospective and prospective studies, Iseri et al. assessed the diagnostic accuracy of the UTI-lizer (digital dipstick), a culture-based detection POC device developed by UTIlizer AB, Stockholm, Sweden, that aids in the rapid identification and quantification of five distinct bacteria in the urine in under two minutes. The 180 microculture wells are filled with a three-step process that involves inserting the dipstick in urine to inoculate the sample, incubating it at 37°C, and then analysing the digital image with a smartphone or scanner in which the colour change indicates a positive result. Compared to clinical standards, the retrospective study demonstrated 100% sensitivity and specificity in detecting bacteriuria, 98.6% sensitivity and 96.8% specificity in identifying primary pathogens with UTI-lizer. Meanwhile, the prospective study showed 100% sensitivity and 89.6% specificity in detecting significant bacteriuria for in-panel microorganisms (Iseri et al., 2024).

## 4 Treatment and management of UTI

The antimicrobial usage guidelines published by IDSA, offer valuable information to medical professionals that can be used directly or serve as the foundation for the hospital guidelines on the treatment of a range of infectious disorders, including urinary tract infections (Nicolle et al., 2005; Weese et al., 2011). The National Institute for Health and Clinical Excellence recommends a course of oral antibiotics for a brief period of 7-10 days for acute UTIs, while it is 10 -14 days for pyelonephritis (Neu, 1992a; Novelli and Rosi, 2017 for effective treatment to prevent antimicrobial resistance and recurrence of the infection (Neu, 1992; Novelli and Rosi, 2017). Empirical antimicrobial treatment with broad-spectrum antibiotics is initiated experimentally prior to the availability of urine culture results considering factors such as patient characteristics (comorbidities, allergies, concurrent medication, and compliance), regional practice patterns, the incidence of resistance in the local community, product availability, and price (Neu, 1992; Novelli and Rosi, 2017; Alkhawaldeh et al., 2022; Ara et al., 2022).

Since 1940s, antimicrobial medicines have been utilized to treat UTI. The first-line treatment with nitrofurantoin, trimethoprim-sulfamethoxazole, fosfomycin trometamol, β-lactam medicines, and fluoroquinolones, and cephalosporins including cefuroxime, axetil, and cefixime are the preferred medications for uncomplicated UTIs (Jancel, 2002). Compared to nitrofurantoin and β -lactams, TMP and fluoroquinolones produce large quantities of vaginal secretions that might be more effective to completely remove uropathogens from the vagina (Nicolle, 2003).

Vellinga et al. showed that 56% of the patients received a prescription for an antimicrobial medication, while only 14% of them had the isolate identified. Trimethoprim, nitrofurantoin, fluoroquinolones, or co-amoxyclav, were commonly used for uncomplicated UTIs, but trimethoprim alone as an empirical treatment was less successful due to the increased resistance (Vellinga et al., 2011).

The study by Alkhawaldeh et al. showed that there was no correlation between the culture results and the treatment that could lead to antimicrobial resistance. The statistics of their study showed that out of 87.6% patients who got empirical treatment, only 10.5% of patients saw improvement in their management of UTIs once culture and sensitivity findings were known, while 0.8% of patients saw a worsening of their therapy (Alkhawaldeh et al., 2022). Studies shows that urine isolates are susceptible to fosfomycin, nitrofurantoin, amoxiclav, and meropenem and can be used to treat lower UTI (Sharmin et al., 2022 (Sharma et al., 2021). Pregnancy induces several physiological, hormonal, and functional changes in the urinary tract, demanding the need for urine culture. During pregnancy physiological hormonal and functional changes demand for the proper selection of antibiotic, with FDA category B antimicrobials such as penicillins, oral cephalosporins, and fosfomycin trometamol are advised for the lower urinary tract infections while parenteral cephalosporins, penicillins, or monobactams (aztreonam) are preferred for acute pyelonephritis. Some antibiotics can have effects like dysplasia and discolouration of teeth and bones, restrict the development of the neural tube, hemolysis and glucose-6-phosphate dehydrogenase (G6PD) impairment, obstruct the eighth nerve in foetus, and changes in the joint cartilages respectively (Krcmery et al., 2001) (**Figure 2**).

Synthetic and nylon innerwear increases UTI risk as they do not absorb sweat as cotton innerwear leading the urogenital area wet and prone to infection (Vyas, 2015). Hence, maintaining proper perineal cleanliness i.e., cleaning from genitalia to the anus, changing the soiled sanitary pad frequently needs to be considered (Das et al., 2015). According to a study by Jelly et al. there were fewer UTIs when women used peri-wash more frequently after urinating and during their menstrual cycle (Jelly et al., 2022).

Cranberries, in its various form, is used as prophylactic measure to treat lower and recurrent UTIs has no scientific evidence for therapeutics. The presence of various phytochemicals, especially, quinic acid and anthocyanidin/proanthocyanidin are thought to cause the urine to contain high levels of hippuric acid, triggering antibacterial properties and prevent type I and P-fimbriated uropathogens to adhere to the bladder wall (Kinney and Blount, 1979; Guay, 2009; Hisano et al., 2012; Jepson et al., 2012).

Probiotics, particularly *lactobacilli* spp., replenish the urogenital flora disrupted by antibiotics thus prevent UTIs. (Falagas et al., 2006; Akgul and Karakan, 2018) (Falagas et al., 2006 Lactin V (Osel), contains hydrogen peroxide-producing (H_2_O_2_^+^) *Lactobacillus crispatus* strain CTV-05, which aids women who are susceptible to UTIs to regain a normal vaginal flora with treatment leading to an intense and protracted colonization with *Lactobacillus crispatus* in women with rUTI (Stapleton et al., 2011).

## 5 Conclusion

UTI is one of the most common infectious diseases affecting women at a higher rate due to their anatomy and physiology and increases the socio-economic burden of society. Early disease diagnosis is crucial to combat AMR organisms. There are a large number of predisposing and lifestyle-related factors that lead to UTIs. The current diagnostic techniques are mainly culture tests and dipstick analysis, which are time-consuming. The emergence of user-friendly, cost-effective, and adaptable diagnostic approaches, including paper-based, electrochemical, and optical sensors, holds promise for early UTI detection. The use of bacterial biomarkers helps in the diagnosis, as the individual biomarkers can be affected by the immune condition. Also, medical professionals prescribe antibiotics for the treatment of UTIs, but proper diagnosis and treatment of UTIs are required to counter the infection. Proper hygiene, using plant sources like cranberry, and consuming probiotics effectively prevent UTIs. By embracing these advancements and working together to implement preventive measures, we can take a more comprehensive approach to tackling the challenges associated with UTIs.

## References

[B1] Abdelaal A. M. Salah A. A. Alhamshary A. Saad O. Shaer E. Mohamedyounes A. Z.. (2019).Urinary Interleukin-6 as biomarker for diagnosis of acute pyelonephritis in children. Available online at: https://geget.journals.ekb.eg/.

[B2] Abed H. A. Mohammed Ali K. O. Alrifai S. B. (2021). Estimation of IL-6 and IL-8 in the urine and serum of children with urinary tract infection. Indian J. Forensic Med. Toxicol. 15. doi: 10.37506/ijfmt.v15i3

[B3] Acton S. O’Meara Y. M. (1997). Urinary tract infection and contraceptive method. Irish Med. J. 90, 176. 9345824

[B4] Adegun P. T. Odimayo M. S. Olaogun J. G. Emmanuel E. E. (2019). Comparison of uropathogens and antibiotic susceptibility patterns in catheterized ambulant middle-aged and elderly Nigerian patients with bladder outlet obstruction. Türk Üroloji Dergisi/Turkish J. Urol. 45, 48–55. doi: 10.5152/tud.2018.25588 PMC6342574 29975632

[B5] Adrover-Jaume C. Rojo-Molinero E. Clemente A. Russell S. M. Arranz J. Oliver A.. (2020). Mobile origami immunosensors for the rapid detection of urinary tract infections. Anal. 145, 7916–7921. doi: 10.1039/D0AN01218A 33020772

[B6] Agrawal P. Pandey A. Sompura S. Pursnani M. L. (2013). Role of blood C - reactive protein levels in upper urinary tract infection and lower urinary tract infection in adult patients (>16 years). J. Assoc. Phys. India 61, 462–463. 24772749

[B7] Akgul T. Karakan T. (2018). The role of probiotics in women with recurrent urinary tract infections. Türk Üroloji Dergisi/Turkish J. Urol. 44, 377–383. doi: 10.5152/tud.2018.48742 PMC6134985 30487041

[B8] Akhtar A. Ahmad Hassali M. A. Zainal H. Ali I. Khan A. H. (2021). A cross-sectional assessment of urinary tract infections among geriatric patients: prevalence, medication regimen complexity, and factors associated with treatment outcomes. Front. Public Health 9. doi: 10.3389/fpubh.2021.657199 PMC8558341 34733812

[B9] Alkhawaldeh R. Abu Farha R. Abu Hammour K. Alefishat E. (2022). Optimizing antimicrobial therapy in urinary tract infections: A focus on urine culture and sensitivity testing. Front. Pharmacol. 13. doi: 10.3389/fphar.2022.1058669 PMC9748416 36532780

[B10] Allison J. S. Dawson M. Drake D. Montie T. C. (1985). Electrophoretic separation and molecular weight characterization of *Pseudomonas aeruginosa* H-antigen flagellins. Infect. Immun. 49, 770–774. doi: 10.1128/iai.49.3.770-774.1985 3928494 PMC261271

[B11] Al Rushood M. AL-Eisa A. AL-Attiyah R. (2020). Serum and urine interleukin-6 and interleukin-8 levels do not differentiate acute pyelonephritis from lower urinary tract infections in children. J. Inflammation Res. 13, 789–797. doi: 10.2147/JIR.S275570 PMC7604446 33149653

[B12] Alshomrani M. K. Alharbi A. A. Alshehri A. A. Arshad M. Dolgum S. (2023). Isolation of *Staphylococcus aureus* Urinary Tract Infections at a Community-Based Healthcare Center in Riyadh. Cureus. 15 (2), e35140. doi: 10.7759/cureus.35140 36949976 PMC10027110

[B13] Amdekar S. Singh V. Singh D. D. (2011). Probiotic therapy: immunomodulating approach toward urinary tract infection. Curr. Microbiol. 63, 484–490. doi: 10.1007/s00284-011-0006-2 21901556

[B14] Andriole V. T. (1987). Urinary tract infections: recent developments. J. Infect. Dis. 156, 865–869. Available at: https://about.jstor.org/terms. 3316414 10.1093/infdis/156.6.865

[B15] Ara R. Mohammad Nasrullah S. Tasnim Z. Afrin S. Saif-Ur-Rahman K. M. Hawlader M. D. H. (2022). Effective antimicrobial therapies of urinary tract infection among children in low-income and middle-income countries: protocol for a systematic review and meta-analysis. BMJ Open 12, e060568. doi: 10.1136/bmjopen-2021-060568 PMC9006796 35414563

[B16] Arabi Z. Al Thiab K. Altheaby A. Aboalsamh G. Kashkoush S. Almarastani M.. (2021). Urinary tract infections in the first 6 months after renal transplantation. Int. J. Nephrol. 2021, 1–8. doi: 10.1155/2021/3033276 PMC8608522 34820141

[B17] Arao S. Matsuura S. Nonomura M. Miki K. Kabasawa K. Nakanishi H. (1999). Measurement of urinary lactoferrin as a marker of urinary tract infection. J. Clin. Microbiol. 37, 553–557. doi: 10.1128/JCM.37.3.553-557.1999 9986811 PMC84465

[B18] Arienzo A. Cellitti V. Ferrante V. Losito F. Stalio O. Murgia L.. (2020). A new point-of-care test for the rapid detection of urinary tract infections. Eur. J. Clin. Microbiol. Infect. Dis. 39, 325–332. doi: 10.1007/s10096-019-03728-3 31707506 PMC7010689

[B19] Arinzon Z. Peisakh A. Shuval I. Shabat S. Berner Y. N. (2009). Detection of urinary tract infection (UTI) in long-term care setting: Is the multireagent strip an adequate diagnostic tool? Arch. Gerontol. Geriat. 48, 227–231. doi: 10.1016/j.archger.2008.01.012 18314207

[B20] Aubais aljelehawy Q. Hadi Alshaibah L. H. Abbas Al- Khafaji Z. K. (2021). Evaluation of virulence factors among *Staphylococcus aureus* strains isolated from patients with urinary tract infection in Al-Najaf Al-Ashraf teaching hospital. Cell. Mol. Biomed. Rep. 1, 78–87. doi: 10.55705/cmbr.2021.144995.1017

[B21] Baba-Moussa L. Anani L. Scheftel J. M. Couturier M. Riegel P. Haïkou N.. (2008). Virulence factors produced by strains of *Staphylococcus aureus* isolated from urinary tract infections. J. Hosp. Infect. 68, 32–38. doi: 10.1016/j.jhin.2007.10.010 18069084

[B22] Bacârea A. Fekete G. Grigorescu B. Bacârea V. (2021). Discrepancy in results between dipstick urinalysis and urine sediment microscopy. Exp. Ther. Med. 21, 538. doi: 10.3892/etm.2021.9971 33815611 PMC8014952

[B23] Baldrich E. Muñoz F. X. García-Aljaro C. (2011). Electrochemical detection of quorum sensing signaling molecules by dual signal confirmation at microelectrode arrays. Anal. Chem. 83, 2097–2103. doi: 10.1021/ac1028243 21323339

[B24] Balogh E. P. Miller B. T. Ball J. R. (Eds.) (2015). Improving Diagnosis in Health Care (Washington (DC): National Academies Press). doi: 10.17226/21794 26803862

[B25] Bardsley A. (2017). Diagnosis, prevention and treatment of urinary tract infections in older people. Nurs. Older People 29, 32–38. doi: 10.7748/nop.2017.e884 28244347

[B26] Belyayeva M. Jeong J. M. (2023). Acute pyelonephritis. 30137822

[B27] Bethel J. (2012). Acute pyelonephritis: risk factors, diagnosis and treatment. Nurs. Stand. 27, 51–56. doi: 10.7748/ns2012.10.27.5.51.c9334 23256302

[B28] Beyer A. K. Currea G. C. C. Holm A. (2019). Validity of microscopy for diagnosing urinary tract infection in general practice – a systematic review. Scandinavian J. Prim. Health Care 37, 373–379. doi: 10.1080/02813432.2019.1639935 PMC6713105 31304845

[B29] Bijlsma I. G. W. Dijk L. Kusters J. G. Gaastra W. (1995). Nucleotide sequences of two fimbrial major subunit genes, *pmpA* and *ucaA*, from canine-uropathogenic *Proteus mirabilis* strains. Microbiology 141, 1349–1357. doi: 10.1099/13500872-141-6-1349 7670636

[B30] Bjerklund Johansen T. E. (2004). The role of imaging in urinary tract infections. World J. Urol. 22, 392–398. doi: 10.1007/s00345-004-0414-z 15290204

[B31] Blom M. Sørensen T. L. Espersen F. Frimodt-Møller N. (2002). Validation of FLEXICULTTM SSI-urinary kit for use in the primary health care setting. Scandinavian J. Infect. Dis. 34, 430–435. doi: 10.1080/00365540110080601 12160170

[B32] Bottini G. (2013). A new method for microbiological analysis that could be used for point-of-care testing (POCT). Open Emergency Med. J. 5, 13–15. doi: 10.2174/1876542401305010013

[B33] Burgoyne E. D. Molina-Osorio A. F. Moshrefi R. Shanahan R. McGlacken G. P. Stockmann T. J.. (2020). Detection of *Pseudomonas aeruginosa* quorum sensing molecules at an electrified liquid|liquid micro-interface through facilitated proton transfer. Anal. 145, 7000–7008. doi: 10.1039/D0AN01245A 32869782

[B34] Buzid A. Shang F. Reen F. J. Muimhneacháin E.Ó. Clarke S. L. Zhou L.. (2016). Molecular Signature of *Pseudomonas aeruginosa* with Simultaneous Nanomolar Detection of Quorum Sensing Signaling Molecules at a Boron-Doped Diamond Electrode. Sci. Rep. 6, 30001. doi: 10.1038/srep30001 27427496 PMC4948026

[B35] Califf R. M. (2018). Biomarker definitions and their applications. Exp. Biol. Med. 243, 213–221. doi: 10.1177/1535370217750088 PMC5813875 29405771

[B36] Capatina D. Lupoi T. Feier B. Blidar A. Hosu O. Tertis M.. (2022). Label-free electrochemical aptasensor for the detection of the 3-O-C12-HSL quorum-sensing molecule in *pseudomonas aeruginosa* . Biosensors 12, 440. doi: 10.3390/bios12070440 35884243 PMC9312901

[B37] Caretto M. Giannini A. Russo E. Simoncini T. (2017). Preventing urinary tract infections after menopause without antibiotics. Maturitas 99, 43–46. doi: 10.1016/j.maturitas.2017.02.004 28364867

[B38] Chen S. L. Jackson S. L. Boyko E. J. (2009). Diabetes mellitus and urinary tract infection: epidemiology, pathogenesis and proposed studies in animal models. J. Urol. 182, S51–6. doi: 10.1016/j.juro.2009.07.090 19846134

[B39] Chien T.-I. Lu J.-Y. Kao J.-T. Lee T.-F. Ho S.-Y. Chang C.-Y.. (2007). Comparison of three automated urinalysis systems—Bayer Clinitek Atlas, Roche Urisys 2400 and Arkray Aution Max for testing urine chemistry and detection of bacteriuria. Clinica Chimica Acta 377, 98–102. doi: 10.1016/j.cca.2006.08.033 17049339

[B40] Chowdhury E. Zubair A. (2022). Triangular gold nanoplates integrated microgel-based sensor for urinary tract infection and glucosuria detection. Opt. Mater. Express 12, 2212. doi: 10.1364/OME.456759

[B41] Chu C. M. Lowder J. L. (2018). Diagnosis and treatment of urinary tract infections across age groups. Am. J. Obstet. Gynecol. 219, 40–51. doi: 10.1016/j.ajog.2017.12.231 29305250

[B42] Chua A. Yean C. Y. Ravichandran M. Lim B. Lalitha P. (2011). A rapid DNA biosensor for the molecular diagnosis of infectious disease. Biosensors Bioelectron. 26, 3825–3831. doi: 10.1016/j.bios.2011.02.040 21458979

[B43] Clark L. C. Lyons C. (1962). Electrode systems for continuous monitoring in cardiovascular surgery. Ann. New York Acad. Sci. 102, 29–45. doi: 10.1111/j.1749-6632.1962.tb13623.x 14021529

[B44] Colella M. Topi S. Palmirotta R. D’Agostino D. Charitos I. A. Lovero R.. (2023). An overview of the microbiota of the human urinary tract in health and disease: current issues and perspectives. Life 13, 1486. doi: 10.3390/life13071486 37511861 PMC10381901

[B45] Dalet F. Segovia T. (1995). Evaluation of a new agar in Uricult-Trio for rapid detection of *Escherichia coli* in urine. J. Clin. Microbiol. 33, 1395–1398. doi: 10.1128/jcm.33.5.1395-1398.1995 7615766 PMC228177

[B46] Das P. Baker K. K. Dutta A. Swain T. Sahoo S. Das B. S.. (2015). Menstrual hygiene practices, WASH access and the risk of urogenital infection in women from Odisha, India. PloS One 10, e0130777. doi: 10.1371/journal.pone.0130777 26125184 PMC4488331

[B47] Davis N. F. Flood H. D. (2011). “The Pathogenesis of Urinary Tract Infections,” in Clinical Management of Complicated Urinary Tract Infection (InTech). doi: 10.5772/22308

[B48] Davoudabadi S. Goudarzi M. Hashemi A. (2023). Detection of Virulence Factors and Antibiotic Resistance among *Klebsiella pneumoniae* Isolates from Iran. BioMed. Res. Int. 2023, 1–7. doi: 10.1155/2023/3624497 PMC9943618 36825037

[B49] Deb A. Gogoi M. Mandal T. K. Sinha S. Pattader P. S. G. (2023). Specific instantaneous detection of *klebsiella pneumoniae* for UTI diagnosis with a plasmonic gold nanoparticle conjugated aptasensor. ACS Appl. Bio Mater. 6, 3309–3318. doi: 10.1021/acsabm.3c00369 37437266

[B50] de Paiva-Santos W. de Sousa V. S. Giambiagi-deMarval M. (2018). Occurrence of virulence-associated genes among *Staphylococcus saprophyticus* isolated from different sources. Microbial. Pathogen. 119, 9–11. doi: 10.1016/j.micpath.2018.03.054 29604423

[B51] Dienye P. O. Gbeneol P. K. (2011). Contraception as a risk factor for urinary tract infection in Port Harcourt, Nigeria: A case control study. Afr. J. Prim. Health Care Family Med. 3, 1–4. doi: 10.4102/phcfm.v3i1.207

[B52] Dutta C. Pasha K. Paul S. Abbas M. S. Nassar S. T. Tasha T.. (2022). Urinary tract infection induced delirium in elderly patients: A systematic review. Cureus. 14 (12), e32321. doi: 10.7759/cureus.32321 36632270 PMC9827929

[B53] Ebrahimzadeh T. Kuprasertkul A. Neugent M. L. Lutz K. C. Fuentes J. L. Gadhvi J.. (2021). Urinary prostaglandin E2 as a biomarker for recurrent UTI in postmenopausal women. Life Sci. Alliance 4, e202000948. doi: 10.26508/lsa.202000948 33958485 PMC8200289

[B54] Elkhawaga A. A. Khalifa M. M. El-badawy O. Hassan M. A. El-Said W. A. (2019). Rapid and highly sensitive detection of pyocyanin biomarker in different *Pseudomonas aeruginosa* infections using gold nanoparticles modified sensor. PloS One 14, e0216438. doi: 10.1371/journal.pone.0216438 31361746 PMC6667159

[B55] El-Refaey A. Hagar A. Abo El kheir N. Zeid M. (2020). Diagnostic value of urinary heparin binding protein in urinary tract infection in children. GEGET 15, 10–16. doi: 10.21608/geget.2020.19165.1010

[B56] Falagas M. E. Betsi G. I. Tokas T. Athanasiou S. (2006). Probiotics for prevention of recurrent urinary tract infections in women. Drugs 66, 1253–1261. doi: 10.2165/00003495-200666090-00007 16827601

[B57] Fallahian M. Mashhady E. Amiri Z. (2005). Female urology asymptomatic bacteriuria in users of intrauterine devices. Urol. J. UNRC/IUA 2, 157–159. doi: 10.22037/uj.v2i3.240 17602420

[B58] Fatah M. Waheda N. Kok D. (2016). Detection of urinary lactoferrin as an indicator of urinary tract infection in girls. Zanco J. Med. Sci. 20, 1485–1489. doi: 10.15218/zjms.2016.0048

[B59] Fazly Bazzaz B. S. Darvishi Fork S. Ahmadi R. Khameneh B. (2021). Deep insights into urinary tract infections and effective natural remedies. Afr. J. Urol. 27, 6. doi: 10.1186/s12301-020-00111-z

[B60] Ferreira L. Sánchez-Juanes F. González-Ávila M. Cembrero-Fuciños D. Herrero-Hernández A. González-Buitrago J. M.. (2010). Direct identification of urinary tract pathogens from urine samples by matrix-assisted laser desorption ionization-time of flight mass spectrometry. J. Clin. Microbiol. 48, 2110–2115. doi: 10.1128/JCM.02215-09 20392910 PMC2884468

[B61] Fihn S. D. Latham R. H. Roberts P. Running K. Stamm W. E. (1985). Association between diaphragm use and urinary tract infection. JAMA 254, 240–245. doi: 10.1001/jama.1985.03360020072027 3999367

[B62] Flores-Mireles A. L. Walker J. N. Caparon M. Hultgren S. J. (2015). Urinary tract infections: epidemiology, mechanisms of infection and treatment options. Nat. Rev. Microbiol. 13, 269–284. doi: 10.1038/nrmicro3432 25853778 PMC4457377

[B63] Fogazzi G. B. Grignani S. Colucci P. (1998). Urinary microscopy as seen by nephrologists. Cclm 36, 919–924. doi: 10.1515/CCLM.1998.159 9915223

[B64] Foxman B. (2002). Epidemiology of urinary tract infections: incidence, morbidity, and economic costs. Am. J. Med. 113, 5–13. doi: 10.1016/S0002-9343(02)01054-9 12113866

[B65] Foxman B. (2014). Urinary tract infection syndromes. Infect. Dis. Clinics North America 28, 1–13. doi: 10.1016/j.idc.2013.09.003 24484571

[B66] Fu X.-H. Zhou W. Zhang X.-M. Yin Y.-B. Jing C.-M. Liu L.. (2013). Clinical analysis of 22 cases community-acquired *Pseudomonas aeruginosa* urinary tract infection. Zhonghua Er Ke Za Zhi = Chin. J. Pediatr. 51, 298–301. 23927805

[B67] Gama C. R. B. Pombo M. A. G. Nunes C. P. Gama G. F. Mezitis S. G. Suchmacher Neto M.. (2020). Treatment of recurrent urinary tract infection symptoms with urinary antiseptics containing methenamine and methylene blue: analysis of etiology and treatment outcomes. Res. Rep. Urol. 12, 639–649. doi: 10.2147/RRU.S279060 33365282 PMC7751791

[B68] Ganguly A. Ebrahimzadeh T. Zimmern P. E. De Nisco N. J. Prasad S. (2021). Label free, lateral flow prostaglandin E2 electrochemical immunosensor for urinary tract infection diagnosis. Chemosensors 9, 271. doi: 10.3390/chemosensors9090271

[B69] Ganguly A. Ebrahimzadeh T. Zimmern P. De Nisco N. J. Prasad S. (2022). Label-free, novel electrofluidic capacitor biosensor for prostaglandin E2 detection toward early and rapid urinary tract infection diagnosis. ACS Sensors 7, 186–198. doi: 10.1021/acssensors.1c01951 34928577

[B70] Garcia P. J. You P. Fridley G. Mabey D. Peeling R. (2015). Point-of-care diagnostic tests for low-resource settings. Lancet Global Health 3, e257–e258. doi: 10.1016/S2214-109X(15)70089-6 25889467

[B71] Garibaldi R. A. Burke J. P. Britt M. R. Miller W. A. Smith C. B. (1980). Meatal colonization and catheter-associated bacteriuria. New Engl. J. Med. 303, 316–318. doi: 10.1056/NEJM198008073030605 6991947

[B72] Gavazzi G. Krause K.-H. (2002). Ageing and infection. Lancet Infect. Dis. 2, 659–666. doi: 10.1016/S1473-3099(02)00437-1 12409046

[B73] Gedikbaşı A. Şevketoğlu E. Karyağar S. Sağlampınar Karyağar S. Hatipoğlu S. S. Yılmaz A. (2020). Urine interleukin-1β Can be used for early prediction of urinary tract infection in children. J. Child 20, 1–6. doi: 10.26650/jchild.2020.1.0001

[B74] Giessing M. (2012). Urinary tract infection in renal transplantation. Arab J. Urol. 10, 162–168. doi: 10.1016/j.aju.2012.01.005 26558020 PMC4442899

[B75] Golińska E. (2013). Virulence factors of *Enterococcus* strains isolated from patients with inflammatory bowel disease. World J. Gastroenterol. 19, 3562. doi: 10.3748/wjg.v19.i23.3562 23801857 PMC3691038

[B76] Gomez-Cruz J. Nair S. Manjarrez-Hernandez A. Gavilanes-Parra S. Ascanio G. Escobedo C. (2018). Cost-effective flow-through nanohole array-based biosensing platform for the label-free detection of uropathogenic E. coli real time Biosensors Bioelectron. 106, 105–110. doi: 10.1016/j.bios.2018.01.055 29414075

[B77] Goudarzi M. Mohammadi A. Amirpour A. Fazeli M. Nasiri M. J. Hashemi A.. (2019). Genetic diversity and biofilm formation analysis of *Staphylococcus aureus* causing urinary tract infections in Tehran, Iran. J. Infect. Develop. Countries 13, 777–785. doi: 10.3855/jidc.11329 32074086

[B78] Guay D. R. P. (2009). Cranberry and urinary tract infections. Drugs 69, 775–807. doi: 10.2165/00003495-200969070-00002 19441868

[B79] Habak P. J. Griggs J. R. P. (2023). Urinary tract infection in pregnancy. 30725732

[B80] Hagberg L. Briles D. E. Edén C. S. (1985). Evidence for separate genetic defects in C3H/HeJ and C3HeB/FeJ mice, that affect susceptibility to gram-negative infections. J. Immunol. 134, 4118–4122. doi: 10.4049/jimmunol.134.6.4118 3886795

[B81] Haghi F. Lohrasbi V. Zeighami H. (2019). High incidence of virulence determinants, aminoglycoside and vancomycin resistance in *enterococci* isolated from hospitalized patients in Northwest Iran. BMC Infect. Dis. 19, 744. doi: 10.1186/s12879-019-4395-3 31455296 PMC6712822

[B82] Hansen W. L. J. van der Donk C. F. M. Bruggeman C. A. Stobberingh E. E. Wolffs P. F. G. (2013). A real-time PCR-based semi-quantitative breakpoint to aid in molecular identification of urinary tract infections. PloS One 8, e61439. doi: 10.1371/journal.pone.0061439 23626685 PMC3634083

[B83] Hasan T. H. Alasedi K. K. Jaloob A. A. (2021). Proteus mirabilis virulence factors. International Journal of Pharmaceutical Research 13 (1), 2145–2149. doi: 10.31838/ijpr/2021.13.01.169

[B84] Harris M. Fasolino T. (2022). New and emerging technologies for the diagnosis of urinary tract infections. J Lab Med. 46 (1), 3–15. doi: 10.1515/labmed-2021-0085

[B85] Hashmi S. Kelly E. Rogers S. O. Gates J. (2003). Urinary tract infection in surgical patients. Am. J. Surg. 186, 53–56. doi: 10.1016/S0002-9610(03)00120-X 12842750

[B86] Hayder T. Abusaiba H. Alasedi K. Aljanaby A. (2020). Proteus mirabilis virulence factors: review. Int. J. Pharm. Res. 13 (1), 2145–2149. doi: 10.31838/ijpr/2021.13.01.169

[B87] Heidary Z. Bandani E. Eftekhary M. Jafari A. A. (2016). Virulence genes profile of multidrug resistant *pseudomonas aeruginosa* isolated from Iranian children with UTIs. Acta Med. Iranica 54, 201–210. 27107526

[B88] Herreros M. L. Gili P. del Valle R. Barrios A. Pacheco M. Sánchez A. (2021). Urine collection methods for infants under 3 months of age in clinical practice. Pediatr. Nephrol. 36, 3899–3904. doi: 10.1007/s00467-021-05142-4 34100109

[B89] Hickling D. R. Sun T.-T. Wu X.-R. (2015). Anatomy and physiology of the urinary tract: relation to host defense and microbial infection. Microbiol. Spectr. 3, 1–25. doi: 10.1128/microbiolspec.UTI-0016-2012 PMC4566164 26350322

[B90] Hiraoka M. Hida Y. Hori C. Tsuchida S. Kuroda M. Sudo M. (1995). Urine microscopy on a counting chamber for diagnosis of urinary infection. Pediatr. Int. 37, 27–30. doi: 10.1111/j.1442-200X.1995.tb03680.x 7754761

[B91] Hiraoka M. Hida Y. Tuchida S. Tsukahara H. Yamashita M. Kuroda M.. (1993). Diagnosis of urinary tract infection by urine microscopy using a disposable counting chamber. Scandinavian J. Clin. Lab. Invest. 53, 705–709. doi: 10.3109/00365519309092575 7505946

[B92] Hisano M. Bruschini H. Nicodemo A. C. Srougi M. (2012). Cranberries and lower urinary tract infection prevention. Clinics 67, 661–667. doi: 10.6061/clinics/2012(06)18 22760907 PMC3370320

[B93] Hopkins W. J. Uehling D. T. Wargowski D. S. (1999). Evaluation of a familial predisposition to recurrent urinary tract infections in women. Am. J. Med. Genet. 83, 422–424. doi: 10.1002/(ISSN)1096-8628 10232756

[B94] Horváth J. Wullt B. Naber K. G. Köves B. (2020). Biomarkers in urinary tract infections - which ones are suitable for diagnostics and follow-up? GMS Infect. Dis. 8. doi: 10.3205/id000068 PMC7705555 33299741

[B95] Hsiao C.-Y. Chen T.-H. Lee Y.-C. Hsiao M.-C. Hung P.-H. Wang M.-C. (2020). Risk factors for uroseptic shock in hospitalized patients aged over 80 years with urinary tract infection. Ann. Trans. Med. 8, 477–477. doi: 10.21037/atm.2020.03.95 PMC7210120 32395521

[B96] Ichino M. Kuroyanagi Y. Kusaka M. Mori T. Ishikawa K. Shiroki R.. (2009). Increased urinary neutrophil gelatinase associated lipocalin levels in a rat model of upper urinary tract infection. J. Urol. 181, 2326–2331. doi: 10.1016/j.juro.2009.01.010 19303090

[B97] Iseri E. Nilsson S. van Belkum A. van der Wijngaart W. Özenci V. (2024). Performance of an innovative culture-based digital dipstick for detection of bacteriuria. Microbiol. Spectr. 12, e03613–23. doi: 10.1128/spectrum.03613-23 38088544 PMC10783013

[B98] Jacobsen S. M. Stickler D. J. Mobley H. L. T. Shirtliff M. E. (2008). Complicated Catheter-Associated Urinary Tract Infections Due to *Escherichia coli* and *Proteus mirabilis* . Clin. Microbiol. Rev. 21, 26–59. doi: 10.1128/CMR.00019-07 18202436 PMC2223845

[B99] Jagadesan I. Agarwal I. Chaturvedi S. Jose A. Sahni R. Fleming J. (2019). Urinary neutrophil gelatinase associated lipocalin – A sensitive marker for urinary tract infection in children. Indian J. Nephrol. 29, 340. doi: 10.4103/ijn.IJN_276_18 31571741 PMC6755922

[B100] Jancel T. (2002). Management of uncomplicated urinary tract infections. Western J. Med. 176, 51–55. doi: 10.1136/ewjm.176.1.51 PMC1071654 11788540

[B101] Janev A. Kang J. S. Park S.-Y. (2023). A smartphone integrated paper (SIP)-based platform for rapid and on-site screening of urinary tract infections. Sensors Actuators B: Chem. 382, 133498. doi: 10.1016/j.snb.2023.133498

[B102] Jelly P. Verma R. Kumawat R. Choudhary S. Chadha L. Sharma R. (2022). Occurrence of urinary tract infection and preventive strategies practiced by female students at a tertiary care teaching institution. J. Educ. Health Promo. 11, 122. doi: 10.4103/jehp.jehp_750_21 PMC9170194 35677263

[B103] Jepson R. G. Williams G. Craig J. C. (2012). Cranberries for preventing urinary tract infections. Cochrane Database System. Rev. 2014. doi: 10.1002/14651858.CD001321.pub5 PMC7027998 23076891

[B104] Jia F. Barber E. Turasan H. Seo S. Dai R. Liu L.. (2019). Detection of pyocyanin using a new biodegradable SERS biosensor fabricated using gold coated zein nanostructures further decorated with gold nanoparticles. J. Agric. Food Chem. 67, 4603–4610. doi: 10.1021/acs.jafc.8b07317 30964288

[B105] Jijie R. Kahlouche K. Barras A. Yamakawa N. Bouckaert J. Gharbi T.. (2018). Reduced graphene oxide/polyethylenimine based immunosensor for the selective and sensitive electrochemical detection of uropathogenic Escherichia coli. Sensors Actuators B: Chem. 260, 255–263. doi: 10.1016/j.snb.2017.12.169

[B106] Johansen T. E. B. Botto H. Cek M. Grabe M. Tenke P. Wagenlehner F. M.. (2011). Critical review of current definitions of urinary tract infections and proposal of an EAU/ESIU classification system. Int J Antimicrob Agents. 38, 64–70. doi: 10.1016/j.ijantimicag.2011.09.009 22018988

[B107] John A. S. Mboto C. I. Agbo B. E. (2016). A review on the prevalence and predisposing factors responsible for urinary tract infection among adults. Eur. J. Exp. Biol. 6 (4), 7–11. Available at: https://api.semanticscholar.org/CorpusID:52993535.

[B108] Jover-García J. Gil-Tomás J. J. Díaz-Lantada A. Lafont-Morgado P. Oliver-Sáez P. Colomina-Rodríguez J. (2020). Validación de un dispositivo point-of-care para la detección rápida de infección urinaria y susceptibilidad antimicrobiana. Rev. Chil. Infectol. 37, 523–530. doi: 10.4067/S0716-10182020000500523 33399799

[B109] Jung C. Brubaker L. (2019). The etiology and management of recurrent urinary tract infections in postmenopausal women. Climacteric 22, 242–249. doi: 10.1080/13697137.2018.1551871 30624087 PMC6629580

[B110] Jutel A. (2009). Sociology of diagnosis: a preliminary review. Sociol. Health Ill. 31, 278–299. doi: 10.1111/j.1467-9566.2008.01152.x 19220801

[B111] Kang M. Jo Y. Mun C. Yeom J. Park J. S. Jung H. S.. (2021). Nanoconfined 3D redox capacitor-based electrochemical sensor for ultrasensitive monitoring of metabolites in bacterial communication. Sensors Actuators B: Chem. 345, 130427. doi: 10.1016/j.snb.2021.130427

[B112] Karacan C. Erkek N. Senel S. Akin Gunduz S. Catli G. Tavil B. (2010). Evaluation of urine collection methods for the diagnosis of urinary tract infection in children. Med. Principles Pract. 19, 188–191. doi: 10.1159/000273068 20357500

[B113] Karlsen H. Dong T. (2015). Biomarkers of urinary tract infections: state of the art, and promising applications for rapid strip-based chemical sensors. Anal. Methods 7, 7961–7975. doi: 10.1039/C5AY01678A

[B114] Katongole P. Nalubega F. Florence N. C. Asiimwe B. Andia I. (2020). Biofilm formation, antimicrobial susceptibility and virulence genes of Uropathogenic *Escherichia coli* isolated from clinical isolates in Uganda. BMC Infect. Dis. 20, 453. doi: 10.1186/s12879-020-05186-1 32600258 PMC7325280

[B115] Kaufman J. Temple-Smith M. Sanci L. (2019). Urinary tract infections in children: an overview of diagnosis and management. BMJ Paedia. Open 3, e000487. doi: 10.1136/bmjpo-2019-000487 PMC6782125 31646191

[B116] Kelly B. N. (2023). UTI detection by PCR: Improving patient outcomes. J. Mass Spectrom. Adv. Clin. Lab. 28, 60–62. doi: 10.1016/j.jmsacl.2023.02.006 36895940 PMC9988651

[B117] Kim H. H. Chung M. H. Bin J. H. Cho K. S. Lee J. Suh J.-S. (2018). Urinary YKL-40 as a candidate biomarker for febrile urinary tract infection in young children. Ann. Lab. Med. 38, 39–45. doi: 10.3343/alm.2018.38.1.39 29071817 PMC5700145

[B118] Kinney A. B. Blount M. (1979). Effect of cranberry juice on urinary pH. Nurs. Res. 28, 287–290. doi: 10.1097/00006199-197909000-00012 38439

[B119] Kjölvmark C. Åkesson P. Linder A. (2012). Elevated urine levels of heparin-binding protein in children with urinary tract infection. Pediatr. Nephrol. 27, 1301–1308. doi: 10.1007/s00467-012-2132-x 22410798

[B120] Kjölvmark C. Påhlman L. I. Åkesson P. Linder A. (2014). Heparin-binding protein: A diagnostic biomarker of urinary tract infection in adults. Open Forum Infect. Dis. 1, ofu004. doi: 10.1093/ofid/ofu004 25734078 PMC4324176

[B121] Kogan M. I. Naboka Y. L. Ibishev K. S. Gudima I. A. Naber K. G. (2015). Human urine is not sterile - shift of paradigm. Urol. Internation. 94, 445–452. doi: 10.1159/000369631 25766599

[B122] Konwar A. N. Borse V. (2020). Current status of point-of-care diagnostic devices in the Indian healthcare system with an update on COVID-19 pandemic. Sensors Int. 1, 100015. doi: 10.1016/j.sintl.2020.100015 PMC7280827 34766037

[B123] Krcmery S. Hromec J. Demesova D. (2001). Treatment of lower urinary tract infection in pregnancy. Int. J. Antimicrob. Agents 17, 279–282. doi: 10.1016/S0924-8579(00)00351-4 11295408

[B124] Kumar R. Chhibber S. Gupta V. Harjai K. (2011). Screening & profiling of quorum sensing signal molecules in *Pseudomonas aeruginosa* isolates from catheterized urinary tract infection patients. Indian J. Med. Res. 134, 208–213. 21911974 PMC3181022

[B125] Kumar M. S. Ghosh S. Nayak S. Das A. P. (2016). Recent advances in biosensor based diagnosis of urinary tract infection. Biosensors Bioelectron. 80, 497–510. doi: 10.1016/j.bios.2016.02.023 26890825

[B126] Kumari A. Pasini P. Daunert S. (2008). Detection of bacterial quorum sensing N-acyl homoserine lactones in clinical samples. Anal. Bioanal. Chem. 391, 1619–1627. doi: 10.1007/s00216-008-2002-3 18408921

[B127] Lam C.-W. Law C.-Y. Sze K.-H. To K. K.-W. (2015). Quantitative metabolomics of urine for rapid etiological diagnosis of urinary tract infection: Evaluation of a microbial–mammalian co-metabolite as a diagnostic biomarker. Clinica Chimica Acta 438, 24–28. doi: 10.1016/j.cca.2014.07.038 25108210

[B128] Lehmann L. E. Hauser S. Malinka T. Klaschik S. Weber S. U. Schewe J.-C.. (2011). Rapid qualitative urinary tract infection pathogen identification by septiFast^®^ Real-time PCR. PloS One 6, e17146. doi: 10.1371/journal.pone.0017146 21359187 PMC3040229

[B129] Li R. Leslie S. W. (2023). Cystitis.

[B130] Linder A. Soehnlein O. Åkesson P. (2010). Roles of Heparin-Binding Protein in Bacterial Infections. J Innate Immun. 2 (5), 431–438. doi: 10.1159/000314853 20505311

[B131] Liu Z. Tang H. Xu H. Lu G. Yang W. Xia Z.. (2022). Rapid identification and drug sensitivity test to urinary tract infection pathogens by DOT-MGA. Infect. Drug Resist. 15, 1391–1397. doi: 10.2147/IDR.S356045 35392368 PMC8980293

[B132] Loh K. Sivalingam N. (2007). Urinary tract infections in pregnancy. Malay. Family Physician : Off. J. Acad. Family Phys. Malaysia 2, 54–57. PMC4170332 25606081

[B133] Lubell T. R. Barasch J. M. Xu K. Ieni M. Cabrera K. I. Dayan P. S. (2017). Urinary neutrophil gelatinase–associated lipocalin for the diagnosis of urinary tract infections. Pediatrics 140. doi: 10.1542/peds.2017-1090 PMC6658088 29146619

[B134] Lundstedt A. Leijonhufvud I. Ragnarsdottir B. Karpman D. Andersson B. Svanborg C. (2007). Inherited susceptibility to acute pyelonephritis: A family study of urinary tract infection. J. Infect. Dis. 195, 1227–1234. doi: 10.1086/512620 17357062

[B135] Luppa P. B. Müller C. Schlichtiger A. Schlebusch H. (2011). Point-of-care testing (POCT): Current techniques and future perspectives. TrAC Trends Anal. Chem. 30, 887–898. doi: 10.1016/j.trac.2011.01.019 PMC7125710 32287536

[B136] Mamatha G. (2020). Role of C-reactive protein levels in differentiating upper urinary tract infection and lower urinary tract infection in adults. J. Med. Sci. And Clin. Res. 08, 269–274. doi: 10.18535/jmscr/v8i2.49

[B137] Mambatta A. Jayarajan J. Rashme V. Harini S. Menon S. Kuppusamy J. (2015). Reliability of dipstick assay in predicting urinary tract infection. J. Family Med. Prim. Care 4, 265. doi: 10.4103/2249-4863.154672 25949979 PMC4408713

[B138] Mancuso G. Midiri A. Gerace E. Marra M. Zummo S. Biondo C. (2023). Urinary tract infections: the current scenario and future prospects. Pathogens 12, 623. doi: 10.3390/pathogens12040623 37111509 PMC10145414

[B139] Marmor S. Kerroumi Y. (2016). Patient-specific risk factors for infection in arthroplasty procedure. Orthop. Traumatol.: Surg. Res. 102, S113–S119. doi: 10.1016/j.otsr.2015.05.012 26867708

[B140] Martischang R. Godycki-Ćwirko M. Kowalczyk A. Kosiek K. Turjeman A. Babich T.. (2021). Risk factors for treatment failure in women with uncomplicated lower urinary tract infection. PloS One 16, e0256464. doi: 10.1371/journal.pone.0256464 34464397 PMC8407559

[B141] Mashaly G. E.-S. El-Kazzaz S. S. Zeid M. S. (2020). Urine YKL-40 versus urine NGAL as potential markers for diagnosis of urinary tract infection in febrile pediatric patients. Open J. Immunol. 10, 10–20. doi: 10.4236/oji.2020.101002

[B142] Mazaheri M. (2021). Serum interleukin-6 and interleukin-8 are sensitive markers for early detection of pyelonephritis and its prevention to progression to chronic kidney disease. Int. J. Prev. Med. 12, 2. doi: 10.4103/ijpvm.IJPVM_50_19 34084299 PMC8106277

[B143] Michael I. Kim D. Gulenko O. Kumar S. Kumar S. Clara J.. (2020). A fidget spinner for the point-of-care diagnosis of urinary tract infection. Nat. Biomed. Eng. 4, 591–600. doi: 10.1038/s41551-020-0557-2 32424198

[B144] Miller C. Gilmore J. (2020). Detection of quorum-sensing molecules for pathogenic molecules using cell-based and cell-free biosensors. Antibiotics 9, 259. doi: 10.3390/antibiotics9050259 32429345 PMC7277912

[B145] Montagut E. J. Martin-Gomez M. T. Marco M. P. (2021). An immunochemical approach to quantify and assess the potential value of the pseudomonas quinolone signal as a biomarker of infection. Anal. Chem. 93, 4859–4866. doi: 10.1021/acs.analchem.0c04731 33691411 PMC8479725

[B146] Montagut E. J. Raya J. Martin-Gomez M.-T. Vilaplana L. Rodriguez-Urretavizcaya B. Marco M.-P. (2022). An immunochemical approach to detect the quorum sensing-regulated virulence factor 2-heptyl-4-quinoline N-oxide (HQNO) produced by *pseudomonas aeruginosa* clinical isolates. Microbiol. Spectr. 10, 3237–3246. doi: 10.1128/spectrum.01073-21 PMC9431570 35876587

[B147] Montagut E. J. Vilaplana L. Martin-Gomez M. T. Marco M. P. (2020). High-throughput immunochemical method to assess the 2-heptyl-4-quinolone quorum sensing molecule as a potential biomarker of *pseudomonas aeruginosa* infections. ACS Infect. Dis. 6, 3237–3246. doi: 10.1021/acsinfecdis.0c00604 33210530

[B148] Moon J. H. Yoo K. H. Yim H. E. (2021). Urinary neutrophil gelatinase-associated lipocalin: a marker of urinary tract infection among febrile children. Clin. Exp. Pediatr. 64, 347–354. doi: 10.3345/cep.2020.01130 33091975 PMC8255512

[B149] Muljadi M. Cheng C.-M. Shen C.-J. (2021). Development of a tetrazolium-derived paper-based diagnostic device as an early, alternative bacteria screening tool. Micromachines 13, 44. doi: 10.3390/mi13010044 35056209 PMC8779278

[B150] Narayan Swamy S. N. Jakanur R. K. Sangeetha S. R. (2022). Significance of C-reactive protein levels in categorizing upper and lower urinary tract infection in adult patients. Cureus. 14 (6), e26432. doi: 10.7759/cureus.26432 35915684 PMC9337713

[B151] Naseri M. Halder A. Mohammadniaei M. Prado M. Ashley J. Sun Y. (2021). A multivalent aptamer-based electrochemical biosensor for biomarker detection in urinary tract infection. Electrochimica Acta 389, 138644. doi: 10.1016/j.electacta.2021.138644

[B152] Naseri M. Ziora Z. M. Simon G. P. Batchelor W. (2022). ASSURED-compliant point-of-care diagnostics for the detection of human viral infections. Rev. Med. Virol. 32. doi: 10.1002/rmv.2263

[B153] Neu H. C. (1992). Optimal characteristics of agents to treat uncomplicated urinary tract infections. Infection 20, S266–S271. doi: 10.1007/BF01710012 1294515

[B154] Newman J. W. Floyd R. V. Fothergill J. L. (2017). The contribution of *Pseudomonas aeruginosa* virulence factors and host factors in the establishment of urinary tract infections. FEMS Microbiol. Lett. 364, fnx124. doi: 10.1093/femsle/fnx124 28605563

[B155] Nicolle L. E. (2003). Urinary tract infection: Traditional pharmacologic therapies. Disease-a-Month 49, 111–128. doi: 10.1067/mda.2003.11 12601341

[B156] Nicolle L. E. (2014). Catheter associated urinary tract infections. Antimicrob. Resist. Infect. Control 3, 23. doi: 10.1186/2047-2994-3-23 25075308 PMC4114799

[B157] Nicolle L. E. Bradley S. Colgan R. Rice J. C. Schaeffer A. Hooton T. M. (2005).Infectious diseases society of America guidelines for the diagnosis and treatment of asymptomatic bacteriuria in adults. Available online at: https://about.jstor.org/terms. 10.1086/427507 15714408

[B158] Noiphung J. Laiwattanapaisal W. (2019). Multifunctional paper-based analytical device for *in situ* cultivation and screening of escherichia coli infections. Sci. Rep. 9, 1555. doi: 10.1038/s41598-018-38159-1 30733495 PMC6367442

[B159] Novelli A. Rosi E. (2017). Pharmacological properties of oral antibiotics for the treatment of uncomplicated urinary tract infections. J. Chemother. 29, 10–18. doi: 10.1080/1120009X.2017.1380357 29271734

[B160] Oliveira W. D. Lopes Barboza M. G. Faustino G. Yamanaka Inagaki W. T. Sanches M. S. Takayama Kobayashi R. K.. (2021). Virulence, resistance and clonality of *Proteus mirabilis* isolated from patients with community-acquired urinary tract infection (CA-UTI) in Brazil. Microbial. Pathogen. 152, 104642. doi: 10.1016/j.micpath.2020.104642 PMC7938216 33246088

[B161] Oros D. Ceprnja M. Zucko J. Cindric M. Hozic A. Skrlin J.. (2020). Identification of pathogens from native urine samples by MALDI-TOF/TOF tandem mass spectrometry. Clin. Proteomics 17, 25. doi: 10.1186/s12014-020-09289-4 32581661 PMC7310424

[B162] Pan Y. Sonn G. A. Sin M. L. Y. Mach K. E. Shih M.-C. Gau V.. (2010). Electrochemical immunosensor detection of urinary lactoferrin in clinical samples for urinary tract infection diagnosis. Biosensors Bioelectron. 26, 649–654. doi: 10.1016/j.bios.2010.07.002 PMC2946447 20667707

[B163] Pardeshi P. (2020). Prevalence of urinary tract infections and current scenario of antibiotic susceptibility pattern of bacteria causing UTI. Indian J. Microbiol. Res. 5, 334–338. doi: 10.18231/2394-5478.2018.0070

[B164] Pastells C. Pascual N. Sanchez-Baeza F. Marco M.-P. (2016). Immunochemical determination of pyocyanin and 1-hydroxyphenazine as potential biomarkers of *pseudomonas aeruginosa* infections. Anal. Chem. 88, 1631–1638. doi: 10.1021/acs.analchem.5b03490 26738983

[B165] Pernille H. Lars B. Marjukka M. Volkert S. Anne H. (2019). Sampling of urine for diagnosing urinary tract infection in general practice - First-void or mid-stream urine? Scand J Prim Health Care. 37 (1), 113–119. doi: 10.1080/02813432.2019.1568708 30689471 PMC6452804

[B166] Perrotta C. Aznar M. Mejia R. Albert X. Ng C. W. (2008). Oestrogens for preventing recurrent urinary tract infection in postmenopausal women. Cochrane Database System. Rev. doi: 10.1002/14651858.CD005131.pub2 PMC13598824 18425910

[B167] Price J. R. Guran L. Lim J. Y. Megli C. J. Clark A. L. Edwards S. R.. (2017). Neutrophil gelatinase–associated lipocalin biomarker and urinary tract infections: A diagnostic case-control study (NUTI study). Female Pelvic Med. Reconstruct. Surg. 23, 101–107. doi: 10.1097/SPV.0000000000000366 28106649

[B168] Rafiee M. Ghaemi E. A. (2023). Detection of virulence genes among *Staphylococcus saprophyticus* isolated from women with urinary tract infections: first report from Iran. BMC Res. Notes 16, 206. doi: 10.1186/s13104-023-06481-1 37697340 PMC10496302

[B169] Riccabona M. (2016). Imaging in childhood urinary tract infection. La Radiol. Med. 121, 391–401. doi: 10.1007/s11547-015-0594-1 26530242

[B170] Rosenberg M. Berger S. A. Barki M. Goldberg S. Fink A. Miskin A. (1992). Initial testing of a novel urine culture device. J. Clin. Microbiol. 30, 2686–2691. doi: 10.1128/jcm.30.10.2686-2691.1992 1400968 PMC270499

[B171] Rowe T. A. Juthani-Mehta M. (2013). Urinary tract infection in older adults. Aging Health 9, 519–528. doi: 10.2217/ahe.13.38 PMC3878051 24391677

[B172] Rychert J. (2019). Benefits and limitations of MALDI-TOF mass spectrometry for the identification of microorganisms. J. Infectiol. 2, 1–5. doi: 10.29245/2689-9981/2019/4.1142

[B173] Sahu R. Sahoo R. K. Prusty S. K. Sahu P. K. (2018). Urinary tract infection and its management. System. Rev. Pharm. 10, 42–48. doi: 10.5530/srp.2019.1.7

[B174] Saliba W. Nitzan O. Chazan B. Elias M. (2015). Urinary tract infections in patients with type 2 diabetes mellitus: review of prevalence, diagnosis, and management. Diab. Metab. Syndr. Obes.: Targets Ther. 129, 129–136. doi: 10.2147/DMSO.S51792 PMC4346284 25759592

[B175] Sarshar M. Behzadi P. Ambrosi C. Zagaglia C. Palamara A. T. Scribano D. (2020). FimH and anti-adhesive therapeutics: A disarming strategy against uropathogens. Antibiotics 9, 397. doi: 10.3390/antibiotics9070397 32664222 PMC7400442

[B176] Scarparo C. Piccoli P. Ricordi P. Scagnelli M. (2002). Evaluation of the dipstreak, a new device with an original streaking mechanism for detection, counting, and presumptive identification of urinary tract pathogens. J Clin Microbiol. 40 (6), 2169–2178. doi: 10.1128/JCM.40.6.2169-2178.2002 12037082 PMC130732

[B177] Schmiemann G. Kniehl E. Gebhardt K. Matejczyk M. M. Hummers-Pradier E. (2010). The diagnosis of urinary tract infection. Deutsches Ärzteblatt Int. 107 (21), 361–367. doi: 10.3238/arztebl.2010.0361 PMC2883276 20539810

[B178] Schneeberger C. Kazemier B. M. Geerlings S. E. (2014). Asymptomatic bacteriuria and urinary tract infections in special patient groups. Curr. Opin. Infect. Dis. 27, 108–114. doi: 10.1097/QCO.0000000000000028 24296584

[B179] Scholes D. Hooton T. M. Roberts P. L. Gupta K. Stapleton A. E. Stamm W. E. (2005). Risk factors associated with acute pyelonephritis in healthy women. Ann. Internal Med. 142, 20. doi: 10.7326/0003-4819-142-1-200501040-00008 15630106 PMC3722605

[B180] Seifu W. D. Gebissa A. D. (2018). Prevalence and antibiotic susceptibility of Uropathogens from cases of urinary tract infections (UTI) in Shashemene referral hospital, Ethiopia. BMC Infect. Dis. 18, 30. doi: 10.1186/s12879-017-2911-x 29320984 PMC5763535

[B181] Shah C. Baral R. Bartaula B. Shrestha L. B. (2019). Virulence factors of uropathogenic *Escherichia coli* (UPEC) and correlation with antimicrobial resistance. BMC Microbiol. 19, 204. doi: 10.1186/s12866-019-1587-3 31477018 PMC6720075

[B182] Sharma S. Verma P. K. Rawat V. Varshney U. Singh R. K. (2021). Fosfomycin versus nitrofurantoin for the treatment of lower UTI in outpatients. J. Lab. Phys. 13, 118–122. doi: 10.1055/s-0041-1729141 PMC8409121 34483555

[B183] Sharmin S. Kamal S. M. M. Md. A. Elahi K. M. A. Elma S. M. M. Habib B. (2022). Fosfomycin—A promising oral antibiotic for the treatment of urinary tract infection (UTI). Open J. Urol. 12, 257–270. doi: 10.4236/oju.2022.125026

[B184] Sharp D. Gladstone P. Smith R. B. Forsythe S. Davis J. (2010). Approaching intelligent infection diagnostics: Carbon fibre sensor for electrochemical pyocyanin detection. Bioelectrochemistry 77, 114–119. doi: 10.1016/j.bioelechem.2009.07.008 19666245

[B185] Shaw J. L. V. (2016). Practical challenges related to point of care testing. Pract. Lab. Med. 4, 22–29. doi: 10.1016/j.plabm.2015.12.002 28856189 PMC5574506

[B186] Shih C.-M. Chang C.-L. Hsu M.-Y. Lin J.-Y. Kuan C.-M. Wang H.-K.. (2015). Paper-based ELISA to rapidly detect *Escherichia coli* . Talanta 145, 2–5. doi: 10.1016/j.talanta.2015.07.051 26459436

[B187] Siddiqui H. Nederbragt A. J. Lagesen K. Jeansson S. L. Jakobsen K. S. (2011). Assessing diversity of the female urine microbiota by high throughput sequencing of 16S rDNA amplicons. BMC Microbiol. 11, 244. doi: 10.1186/1471-2180-11-244 22047020 PMC3228714

[B188] Sinawe H. Casadesus D. (2023). Urine culture. 32491501

[B189] Singhal N. Kumar M. Kanaujia P. K. Virdi J. S. (2015). ). MALDI-TOF mass spectrometry: an emerging technology for microbial identification and diagnosis. Front. Microbiol. 6. doi: 10.3389/fmicb.2015.00791 PMC4525378 26300860

[B190] Smelov V. Naber K. Bjerklund Johansen T. E. (2016). Improved classification of urinary tract infection: future considerations. Eur. Urol. Suppl. 15, 71–80. doi: 10.1016/j.eursup.2016.04.002

[B191] Soo Park B. Lee S.-J. Wha Kim Y. Sik Huh J. Il Kim J. Chang S.-G. (2006). Outcome of nephrectomy and kidney-preserving procedures for the treatment of emphysematous pyelonephritis. Scandinavian J. Urol. Nephrol. 40, 332–338. doi: 10.1080/00365590600794902 16916776

[B192] Stapleton A. E. Au-Yeung M. Hooton T. M. Fredricks D. N. Roberts P. L. Czaja C. A.. (2011). Randomized, placebo-controlled phase 2 trial of a *lactobacillus crispatus* probiotic given intravaginally for prevention of recurrent urinary tract infection. Clin. Infect. Dis. 52, 1212–1217. doi: 10.1093/cid/cir183 21498386 PMC3079401

[B193] St-Louis P. (2000). Status of point-of-care testing: promise, realities, and possibilities. Clin. Biochem. 33, 427–440. doi: 10.1016/S0009-9120(00)00138-7 11074234

[B194] Storme O. Tirán Saucedo J. Garcia-Mora A. Dehesa-Dávila M. Naber K. G. (2019). Risk factors and predisposing conditions for urinary tract infection. Ther. Adv. Urol. 11, 175628721881438. doi: 10.1177/1756287218814382 PMC6502981 31105772

[B195] Svetličić E. Dončević L. Ozdanovac L. Janeš A. Tustonić T. Štajduhar A.. (2022). Direct identification of urinary tract pathogens by MALDI-TOF/TOF analysis and *de novo* peptide sequencing. Molecules 27, 5461. doi: 10.3390/molecules27175461 36080229 PMC9457756

[B196] Tabatabaei A. Ahmadi K. Namazi Shabestari A. Khosravi N. Badamchi A. (2021). Virulence genes and antimicrobial resistance pattern in *Proteus mirabilis* strains isolated from patients attended with urinary infections to Tertiary Hospitals, in Iran. Afr. Health Sci. 21, 1677–1684. doi: 10.4314/ahs.v21i4.22 35283944 PMC8889823

[B197] Tabibian J. H. Gornbein J. Heidari A. Dien S. L. Lau V. H. Chahal P.. (2008). Uropathogens and host characteristics. J. Clin. Microbiol. 46, 3980–3986. doi: 10.1128/JCM.00339-08 18842936 PMC2593265

[B198] Tan C. Chlebicki M. (2016). Urinary tract infections in adults. Singapore Med. J. 57, 485–490. doi: 10.11622/smedj.2016153 27662890 PMC5027397

[B199] Tang Y. Zhou Q. (2022). Changes in serum CRP and PCT levels in patients with acute simple lower urinary tract infection and evaluation of the efficacy of treatment with Shuangdong capsules. Emergency Med. Int. 2022, 1–7. doi: 10.1155/2022/9750237 PMC9427299 36052218

[B200] Terlizzi M. E. Gribaudo G. Maffei M. E. (2017). UroPathogenic *escherichia coli* (UPEC) infections: virulence factors, bladder responses, antibiotic, and non-antibiotic antimicrobial strategies. Front. Microbiol. 8. doi: 10.3389/fmicb.2017.01566 PMC5559502 28861072

[B201] Toledo-Arana A. Valle J. Solano C. Arrizubieta M. J. Cucarella C. Lamata M.. (2001). The enterococcal surface protein, esp, is involved in enterococcus faecalis biofilm formation. Appl. Environ. Microbiol. 67, 4538–4545. doi: 10.1128/AEM.67.10.4538-4545.2001 11571153 PMC93200

[B202] Tseng W.-T. Chou Y.-Y. Wu J.-G. Wang Y.-C. Tseng T.-N. Pan S.-W.. (2023). An electrochemical conducting polymer-based biosensor for Leukocyte esterase and nitrite detection for diagnosing urinary tract infections: A pilot study. Microchem. J. 188, 108493. doi: 10.1016/j.microc.2023.108493

[B203] Ünsal H. Kaman A. Tanır G. (2019). Relationship between urinalysis findings and responsible pathogens in children with urinary tract infections. J. Pediatr. Urol. 15, 606.e1–606.e6. doi: 10.1016/j.jpurol.2019.09.017 31735519

[B204] Urbschat A. Obermüller N. Paulus P. Reissig M. Hadji P. Hofmann R.. (2014). Upper and lower urinary tract infections can be detected early but not be discriminated by urinary NGAL in adults. Int. Urol. Nephrol. 46, 2243–2249. doi: 10.1007/s11255-014-0831-x 25218613

[B205] Vasudevan R. (2014). Urinary tract infection: an overview of the infection and the associated risk factors. J. Microbiol. Experiment. 1, 00008. doi: 10.15406/jmen.2014.01.00008

[B206] Vasudevan S. Srinivasan P. Rayappan J. B. B. Solomon A. P. (2020). A photoluminescence biosensor for the detection of N -acyl homoserine lactone using cysteamine functionalized ZnO nanoparticles for the early diagnosis of urinary tract infections. J. Mater. Chem. B 8, 4228–4236. doi: 10.1039/C9TB02243K 32330210

[B207] Vellinga A. Cormican M. Hanahoe B. Bennett K. Murphy A. W. (2011). Antimicrobial management and appropriateness of treatment of urinary tract infection in general practice in Ireland. BMC Family Pract. 12, 108. doi: 10.1186/1471-2296-12-108 PMC3191331 21967276

[B208] Venkatesh L. (2017). Acute pyelonephritis - correlation of clinical parameter with radiological imaging abnormalities. J. Clin. Diagn. Res. TC15–TC18. doi: 10.7860/JCDR/2017/27247.10033 PMC5535453 28764263

[B209] Vyas S. (2015). Role of behavioural risk factors in symptoms related to UTI among nursing students. J. Clin. Diagn. Res. 9 (9), LC15–LC18. doi: 10.7860/JCDR/2015/10995.6547 PMC4606256 26500927

[B210] Wagenlehner F. M. E. Bjerklund Johansen T. E. Cai T. Koves B. Kranz J. Pilatz A.. (2020). Epidemiology, definition and treatment of complicated urinary tract infections. Nat. Rev. Urol. 17, 586–600. doi: 10.1038/s41585-020-0362-4 32843751

[B211] Waisman Y. Zerem E. Amir L. Mimouni M. (1999). The validity of the uriscreen test for early detection of urinary tract infection in children. Pediatrics 104, e41. doi: 10.1542/peds.104.4.e41 10506266

[B212] Wang Y.-C. Tsai Y.-H. Shen C.-F. He M.-Y. Fu Y.-C. Sang C.-Y.. (2021). Turntable paper-based device to detect *escherichia coli* . Micromachines 12, 194. doi: 10.3390/mi12020194 33668560 PMC7917795

[B213] Wang M.-C. Tseng C.-C. Wu A.-B. Lin W.-H. Teng C.-H. Yan J.-J.. (2013). Bacterial characteristics and glycemic control in diabetic patients with Escherichia coli urinary tract infection. J. Microbiol. Immunol. Infect. 46, 24–29. doi: 10.1016/j.jmii.2011.12.024 22572000

[B214] Weese J. S. Blondeau J. M. Boothe D. Breitschwerdt E. B. Guardabassi L. Hillier A.. (2011). Antimicrobial use guidelines for treatment of urinary tract disease in dogs and cats: antimicrobial guidelines working group of the international society for companion animal infectious diseases. Vet. Med. Int. 2011, 1–9. doi: 10.4061/2011/263768 PMC3134992 21776346

[B215] Wen K. Y. Cameron L. Chappell J. Jensen K. Bell D. J. Kelwick R.. (2017). A cell-free biosensor for detecting quorum sensing molecules in P. aeruginosa -infected respiratory samples. ACS Synth. Biol. 6, 2293–2301. doi: 10.1021/acssynbio.7b00219 28981256

[B216] Whelan S. Lucey B. Finn K. (2023). Uropathogenic *escherichia coli* (UPEC)-associated urinary tract infections: the molecular basis for challenges to effective treatment. Microorganisms 11, 2169. doi: 10.3390/microorganisms11092169 37764013 PMC10537683

[B217] Wilksch J. J. Yang J. Clements A. Gabbe J. L. Short K. R. Cao H.. (2011). MrkH, a Novel c-di-GMP-Dependent Transcriptional Activator, Controls *Klebsiella pneumoniae* Biofilm Formation by Regulating Type 3 Fimbriae Expression. PloS Pathog. 7, e1002204. doi: 10.1371/journal.ppat.1002204 21901098 PMC3161979

[B218] Wilson M. L. Gaido L. (2004). Laboratory diagnosis of urinary tract infections in adult patients. Clin. Infect. Dis. 38, 1150–1158. doi: 10.1086/383029 15095222

[B219] Wojno K. J. Baunoch D. Luke N. Opel M. Korman H. Kelly C.. (2020). Multiplex PCR based urinary tract infection (UTI) analysis compared to traditional urine culture in identifying significant pathogens in symptomatic patients. Urology 136, 119–126. doi: 10.1016/j.urology.2019.10.018 31715272

[B220] Wolfe A. J. Toh E. Shibata N. Rong R. Kenton K. FitzGerald M.. (2012). Evidence of uncultivated bacteria in the adult female bladder. J. Clin. Microbiol. 50, 1376–1383. doi: 10.1128/JCM.05852-11 22278835 PMC3318548

[B221] Wu X. Chen J. Li X. Zhao Y. Zughaier S. M. (2014). Culture-free diagnostics of *Pseudomonas aeruginosa* infection by silver nanorod array based SERS from clinical sputum samples. Nanomed.: Nanotechnol. Biol. Med. 10, 1863–1870. doi: 10.1016/j.nano.2014.04.010 PMC4232485 24832961

[B222] Wu P. Zuo W. Wang Y. Yuan Q. Yang J. Liu X.. (2023). Multimodal capture – antibody-independent lateral flow immunoassay based on AuNF – PMBA for point-of-care diagnosis of bacterial urinary tract infections. Chem. Eng. J. 451, 139021. doi: 10.1016/j.cej.2022.139021

[B223] Xu K. Wang Y. Jian Y. Chen T. Liu Q. Wang H.. (2023). Staphylococcus aureus ST1 promotes persistent urinary tract infection by highly expressing the urease. Front. Microbiol. 14. doi: 10.3389/fmicb.2023.1101754 PMC9992547 36910215

[B224] Yamamoto A. Nakayama S. Wakabayashi Y. Yoshino Y. Kitazawa T. (2023). Urine neutrophil gelatinase-associated lipocalin as a biomarker of adult pyelonephritis. J. Infect. Chemother. 29, 508–512. doi: 10.1016/j.jiac.2023.01.001 36621764

[B225] Yang X. Chen H. Zheng Y. Qu S. Wang H. Yi F. (2022). Disease burden and long-term trends of urinary tract infections: A worldwide report. Front. Public Health. 10. doi: 10.3389/fpubh.2022.888205 PMC9363895 35968451

[B226] Yang S. Rothman R. E. (2004). PCR-based diagnostics for infectious diseases: uses, limitations, and future applications in acute-care settings. Lancet Infect. Dis. 4, 337–348. doi: 10.1016/S1473-3099(04)01044-8 15172342 PMC7106425

[B227] Yetisen A. K. Akram M. S. Lowe C. R. (2013). Paper-based microfluidic point-of-care diagnostic devices. Lab. Chip 13, 2210. doi: 10.1039/c3lc50169h 23652632

[B228] Yilmaz A. Sevketoglu E. Gedikbasi A. Karyagar S. Kiyak A. Mulazimoglu M.. (2009). Early prediction of urinary tract infection with urinary neutrophil gelatinase associated lipocalin. Pediatr. Nephrol. 24, 2387–2392. doi: 10.1007/s00467-009-1279-6 19649660

[B229] Yoon B. I. Kim S. W. Ha U.-S. Sohn D. W. Cho Y.-H. (2013). Risk factors for recurrent cystitis following acute cystitis in female patients. J. Infect. Chemother. 19, 727–731. doi: 10.1007/s10156-013-0556-2 23380970

[B230] Yuyun M. F. Angwafo F. F. III Koulla-Shiro S. Zoung-Kanyi J. (2004). Urinary tract infections and genitourinary abnormalities in Cameroonian men. Trop. Med. Int. Health 9, 520–525. doi: 10.1111/j.1365-3156.2004.01219.x 15078271

[B231] Zaffanello M. Malerba G. Cataldi L. Antoniazzi F. Franchini M. Monti E.. (2010). Genetic risk for recurrent urinary tract infections in humans: A systematic review. J. Biomed. Biotechnol. 2010, 1–9. doi: 10.1155/2010/321082 PMC2847765 20379347

[B232] Zeng Z. Zhan J. Zhang K. Chen H. Cheng S. (2022). Global, regional, and national burden of urinary tract infections from 1990 to 2019: an analysis of the global burden of disease study 2019. World J. Urol. 40, 755–763. doi: 10.1007/s00345-021-03913-0 35066637

[B233] Zunino P. (2000). Virulence of a *Proteus mirabilis* ATF isogenic mutant is not impaired in a mouse model of ascending urinary tract infection. FEMS Immunol. Med. Microbiol. 29, 137–143. doi: 10.1016/S0928-8244(00)00198-X 11024353

